# Antibody-conjugated polymer nanoparticles for brain cancer

**DOI:** 10.1007/s13346-025-01947-0

**Published:** 2025-08-20

**Authors:** San San Amelia Tai, Hooi Leong Loo, Athirah Bakhtiar, Paul Chi-Lui Ho, Lay Hong Chuah

**Affiliations:** 1 Division of Biosciences, University College London, Gower Street, WC1E 6BT London, United Kingdom; 2 School of Pharmacy, Monash University Malaysia, Bandar Sunway, Subang Jaya, 47500 Selangor Malaysia

**Keywords:** Brain cancer, Tumor, Antibody, Conjugation, Nanoformulations, Targeting, Targeted therapy

## Abstract

Brain cancer remains a significant global health challenge, affecting over a million individuals worldwide. It includes primary tumors such as gliomas, particularly glioblastoma multiforme (GBM) being one of the most aggressive forms, and secondary brain tumors resulting from metastases of breast or lung cancer. A major obstacle in treating these malignancies is the blood-brain barrier (BBB), which limits effective drug delivery to tumor sites. Nanoparticles have long been explored as versatile drug delivery vehicles due to their potential to improve tumor specificity, penetrate the BBB, and enhance drug bioavailability. To further refine these delivery systems, antibodies have been conjugated to nanoparticles to target specific tumor biomarkers, thereby enhancing therapeutic precision and efficacy. In preclinical studies, antibody-conjugated polymer nanoparticles have demonstrated promising capabilities in achieving both targeted delivery and BBB penetration. This review first outlines the pathophysiology and mechanisms underlying brain cancer. It then discusses how polymer nanoparticles address current therapeutic limitations, particularly in overcoming the BBB and improving pharmacokinetics. Various polymer nanoparticles are explored, including polymeric micelles, dendrimers, nanocapsules, nanospheres, nanogels, and polymersomes, each offering distinct therapeutic advantages. We further examine the impact of surface modification with antibodies to enhance tumor targeting, discussing their physiochemical properties, as well as in vitro and in vivo findings. Finally, the review critically evaluates the current challenges in the development of antibody-conjugated polymer nanoparticles, identifies research gaps, and future directions of this promising field.

**Graphical abstract:**

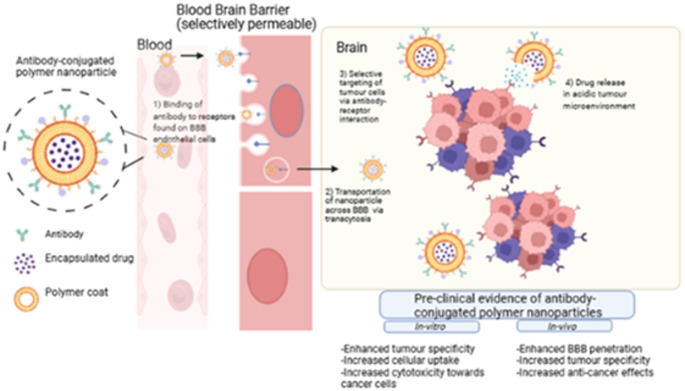

## Introduction

Brain cancer remains one of the most aggressive and least understood malignancies, posing a formidable challenge to modern medicine. Globally, it accounts for a significant burden, with an estimated 1.06 million living with brain cancer in 2019 and approximately 740,000 new cases reported annually [1]. In the UK alone, over 12,000 individuals were diagnosed with a primary brain tumor each year from 2016 to 2018 [2], while the United States projected around 25,400 new cases of brain and other nervous system cancers in 2024 [3]. In Asia, the age-standardized incidence rate (ASIR) for brain and central nervous system cancers was 12.14 per 100,000 individuals in 2019 [4].

Brain cancers comprise a diverse group of primary and secondary neoplasms involving the central nervous system (CNS). Primary brain tumors arise within the CNS and are classified based on their cell or origin and malignancy grade according to the World Health Organization (WHO). These include gliomas, schwannomas, meningiomas, CNS lymphomas, and medulloblastomas. Meningiomas, originating from the meninges (the protective layers of tissue surrounding the brain and spinal cord), are the most prevalent, accounting for approximately 30% of all brain tumors [5, 6]. Medulloblastomas typically affect children and develop in the cerebellum, impacting coordination and balance [7]. Gliomas are a major class of primary brain tumors, encompassing GBM —the most aggressive subtype—as well as astrocytomas and ependymomas. Astrocytomas arise from astrocyte cells and exhibit a wide spectrum of malignancy, whereas ependymomas originate from ependymal cells lining the brain’s ventricles and spinal cord [3]. Schwannomas, usually benign, derive from Schwann cells surrounding cranial nerves, most notably affecting the vestibulocochlear nerve in vestibular schwannoma [5]. Other important but less common CNS tumors include pineal region tumors and primary CNS lymphomas [8]. In contrast, secondary brain tumors, or brain metastases, such as lung, breast or melanoma to the CNS are more common than primary brain tumors [9]. The vast heterogeneity in cellular origin, genetic alterations, and tumor microenvironments among brain cancer subtypes underscores the need for precise, subtype-specific diagnostic and therapeutic strategies [10].

Brain tumors are also classified by grade, based on their aggressiveness. Grade 1 tumors are typically benign and slow-growing, whereas grade 2 tumors, while also slow growing, have the potential to invade nearby tissues [11]. Grade 3 and 4 tumors are highly aggressive and tend to spread rapidly within the brain or to the spinal cord. A single tumor type may an exist across multiple grades and often evolves into a more malignant form over time. Due to the highly malignant nature of grade 3 and 4 tumors, treatments such as radiotherapy and chemotherapy have shown limited efficacy in completely eradicating them [12].

The treatment of brain tumors is further complicated by the presence of the BBB, a selective physiological barrier that protects the brain by restricting the entry of many therapeutic agents [13]. Extensive research has focused on the use of polymer nanoparticles as drug delivery systems capable of traversing the BBB and targeting tumor sites within the brain. These nanoformulations enhance tumor-targeting specificity, promote drug penetration across the BBB, and improve drug bioavailability [14]. To further enhance therapeutic precision, antibody-conjugated polymer nanoparticles have been developed in pre-clinical studies. These systems utilize active ligand-targeting mechanisms, where antibodies act as tumor-directing ligands to direct the nanoparticles to cancer cells. Specific evidence has shown that antibodies, whether used as targeting ligands or therapeutic agents, can inhibit tumor growth more effectively than conventional therapies like chemotherapy [15].

## Brain cancer mechanism

The pathogenesis of brain cancer is primarily driven by genetic mutations that disrupt key molecular signaling pathways responsible for regulating cell growth, differentiation, and death. These mutations impair normal cell cycle control, promote abnormal cellular proliferation, and inhibit programmed cell death, thereby playing a central role in tumor initiation and progression [16]. Advances in gene sequencing technology, such as Sanger sequencing, have enabled more precise classification and diagnosis of brain tumor [17]. In parallel with these technological breakthroughs, researchers have gained deeper insights into the mutational landscape underlying brain cancer. One major discovery is the identification of mutations in the isocitrate dehydrogenase (IDH1/2), commonly found in gliomas, including glioblastoma. The IDH enzymes catalyze the oxidative decarboxylation of isocitrate in the Krebs cycle. Mutations in these genes result in the abnormal production and accumulation of 2-hydroxyglutarate (2HG), a metabolite that promotes oncogenic transformation and tumor progression [18]. Another critical mutation involves the epithelial growth factor receptor (EGFR) gene. In primary glioblastomas, mutations such as EGFRvIII lead to overexpression of EGFR, enhancing cancer cell proliferation, survival and invasiveness by promoting angiogenesis [19]. In medulloblastoma, dysregulation of developmental pathways, particularly Sonic Hedgehog (SHH) pathway, has been shown to contribute significantly to tumor proliferation in medulloblastoma [20]. Additionally, in primary CNS lymphomas, mutations in genes like MYD88 and CD79B result in dysregulation of B-cell receptor signaling, leading to uncontrolled B-cell proliferation within the brain or spinal cord [21].

## Brain cancer treatment strategies

A range of treatment strategies are employed in the management of brain cancer, with the overarching goal of eradicating cancer cells while preserving healthy brain tissue and function. These approaches include surgery, radiation therapy, chemotherapy, immunotherapy, and targeted therapies.

### Surgery

Surgery resection remains the primary treatment for many brain tumors, aiming to remove as much of the tumor mass as possible while minimizing neurological deficits. A common surgical approach is craniotomy, in which the patient is placed under general anesthesia and a section of the skull is temporarily removed to access the tumor. The tumor is then excised using various tools, such as ultrasonic aspiration, surgical scalpel, or microsurgical instruments, depending on its size, location, and histological characteristics [22]. A less invasive alternative is neuroendoscopy, which employs an endoscope to visualize and remove tumor. This technique is particularly advantageous for tumors located in the ventricles or along cerebrospinal fluid pathways, offering shorter recovery time and reduced trauma to surrounding brain tissue [23]. Successful surgery intervention can significantly improve patient survival outcomes and neurological function [24].

### Radiotherapy

Radiotherapy is a widely utilized adjunct or alternative to surgery, employing ionizing radiation, such as X-rays or high-energy proton beams, to destroy cancerous cells, particularly those that are unresectable or infiltrative [25]. Advanced techniques, including three-dimensional conformal radiation therapy (3D-CRT) and proton beam therapy allow for greater precision in targeting tumor margins while sparing tissues [26]. Nonetheless, radiotherapy carries notable limitations and adverse effects. Many high-grade or highly malignant brain tumors exhibit radiation resistance, which diminishes therapeutic efficacy. Additionally, collateral damage to healthy brain tissue can result in cognitive decline or other side effects [27].

### Chemotherapy

Chemotherapy involves the systemic administration of anticancer agents to disrupt various cellular processes and induce apoptosis in cancer cells. Temozolomide (TMZ) is the gold standard chemotherapeutic agent for GBM, working primarily by inducing DNA damage to inhibit cell division [28]. Another agent, tandutinib, is an orally administered, receptor tyrosine kinase inhibitor that targets signaling pathways involved in proliferation and survival, thereby promoting apoptosis in glioma cells [29]. Many chemotherapeutic agents are limited by poor penetration of the BBB and systemic toxicity, underscoring the need for more targeted delivery methods [30].

### Emerging combination therapies

Recent research has increasingly focused on the development of multimodal therapeutic strategies that integrate chemotherapy with complementary approaches such as targeted therapy, immunotherapy, radiotherapy, and nanomedicine. This integrative approach is driven by the limitations of conventional chemotherapy, including drug resistance, systemic toxicity, and limited efficacy in achieving durable tumor control [31]. By leveraging synergistic mechanisms of action, these combination therapies aim to enhance tumor cell eradication, reduce adverse effects, and circumvent resistance pathways, ultimately improving patient survival outcomes across various brain cancer types [32]. For instance, a recent preclinical study investigated the synergistic anti-tumor effects of combining TMZ with niraparib, a poly (ADP-ribose) polymerase (PARP) inhibitor [33]. The combination amplified DNA damage, disrupted DNA repair pathways, induced immunogenic cell death in GBM cells. Notably, it upregulated the expression of UL16-binding protein 1 (ULBP1), a stress-induced ligand that activates natural killer (NK) cells and T cells via the NKG2D receptor pathway. This enhanced expression of ULBP1 facilitated the recognition and elimination of GBM cells by cytotoxic lymphocytes, thereby augmenting anti-tumor immune responses [34]. These findings underscore the potential of combining DNA-damaging agents with immunomodulators to simultaneously exploit tumor-intrinsic vulnerabilities and stimulating immune-mediated clearance of cancer cells [35].

Immune checkpoint inhibitors (ICI), a class of immunotherapies that block regulatory proteins suppressing immune responses, also show a promise in brain cancer therapy [36]. Checkpoint blockade with agents such as nivolumab (anti-PD-1) and ipilimumab (anti-CTLA-4) has been evaluated in combination with TMZ in Phase I clinical trials for newly diagnosed GBM. Importantly, this combination was found to have a manageable safety profile, with toxicity not significantly greater than that observed with monotherapy. Encouraging preliminary outcomes have led to planned Phase II/III trials (NCT number: NCT02311920).

Another emerging strategy involves integrating dendritic cell (DC) vaccines with TMZ to enhance cytotoxic T-cell responses against tumor-specific antigens [37]. In a Phase I clinical study, the safety and preliminary efficacy of autologous DC vaccines administered alongside adjuvant TMZ are being evaluated based on immune activation and patient tolerance. While results are pending (NCT number: NCT04968366), a similar Phase III trial has reported a significant survival benefit among patients with recurrent GBM receiving DCVax-L in combination with TMZ. This group achieved a median survival of 13.2 months compared to 7.8 months in the control group, demonstrating the clinical potential of autologous tumor lysate-loaded DC vaccine as a promising immunochemotherapy approach for brain cancer [38].

### Immunotherapy

Immunotherapy aims to stimulate the patient’s immune system to recognize and eliminate cancer cells. Depending on their mechanism of action, immunotherapeutic strategies are typically categorized into immune checkpoint inhibitors, cancer vaccines, and cytokine-based therapies [39]. The foundational concept of immunotherapy originates from the immune surveillance theory, first proposed in the 1950s, which postulated that the immune system continuously monitors and eliminates emerging tumor cells [40]. This concept laid the groundwork for the development of checkpoint inhibitors, such as anti-CTLA-4 antibodies, which were first approved in 2011 following demonstrations of their efficacy in reactivating T-cells against cancer cells [39].

### Emerging immunotherapeutic strategies

Many immunotherapeutic approaches are currently under preclinical and clinical investigations for their potential applications in brain cancer. These therapies may be used as monotherapies or in combination with conventional treatment such as chemotherapy or radiotherapy [41]. One of the most promising innovations is chimeric antigen receptor (CAR) T-cell therapy, particularly for recurrent high-grade glioma. In contrast to traditional immunotherapies, CAR T-cell therapy involves genetically engineering a patient’s own T cells to express chimeric antigen receptors that recognize and bind to specific antigens on cancer cells [42]. The first CAR T-cell therapy was approved by the FDA in 2017 for the treatment of acute lymphoblastic leukemia [43]. A recent Phase I trial assessed the safety, feasibility and preliminary efficacy of IL-13Rα2-targeted CAR T-cells administered via different routes. Approximately 50% of patients achieved stable disease or better. Nevertheless, therapeutic benefits—including extended survival—was limited to a subset of patients based on the route of administration. These findings emphasize the need for further refinement in administration protocols in future trials to maximize clinical benefits (NCT number: NCT02208362).

Another novel immunotherapeutic strategy involves RNA-lipoplex (RNA-LP) vaccines. A Phase I clinical trial is currently underway to evaluate these vaccines in newly diagnosed adult patients with MGMT unmethylated GBM and pediatric with high-grade gliomas. The primary aims include assessing safety, tolerability, and determining the maximum tolerated dose (MTD) (NCT04573140). These vaccines function by delivering tumor antigen-encoding RNA encapsulated in liposomal carriers to dendritic cells, which then initiate an anti-tumor immune response [44]. RNA-LP vaccines have demonstrated activity in tumors resistant to TMZ and other standard therapies (NCT number: NCT04573140).

Additionally, immune checkpoint inhibitors (ICIs) such as ipilimumab (anti-CTLA-4) and nivolumab (anti-PD-1) have shown potential as neoadjuvant immunotherapies in various solid tumors, including melanoma, non-small cell lung cancer, and triple-negative breast cancer. Although not yet fully explored in brain cancer, these studies provide a basis for investigating ICIs in combination with existing modalities for gliomas and other CNS tumors [45, 46].

Another promising strategy in brain cancer immunotherapy involves the combination of oncolytic viral (OV) therapies with ICIs. OVs exert their anti-tumor effects by selectively infecting and lysing cancer cells through virus-mediated cytotoxicity. This process triggers the release of tumor-associated antigens and pro-inflammatory cytokines, which in turn enhances immune system activation [47]. Additionally, OV therapy has been shown to upregulate PD-L1 expression on tumor cells, thereby increasing their susceptibility to immune checkpoint blockade and improving the overall efficacy of ICIs [41]. While encouraging results have been reported for this combination approach in other cancer types, the therapeutic benefits of neoadjuvant ICIs in patients with GBM remain limited. This is largely attributed to the profoundly immunosuppressive tumor microenvironment of gliomas, their relatively low neoantigen burden, and the restricted permeability of BBB [48].

### Monoclonal antibodies (mAbs)

Monoclonal antibodies (mAbs) are laboratory-engineered molecules designed to mimic the immune system’s ability to recognize and target specific antigens on cancer cells, representing a significant milestone in targeted cancer therapy. These antibodies are produced in large quantities from a single clone, ensuring structural and functional uniformity for therapeutic purposes [49]. A notable example is bevacizumab, a humanized mAb that targets the vascular endothelial receptor (VEGF), a key mediator of angiogenesis and cancer metastasis. Initially approved for colorectal cancer, bevacizumab FDA approval for GBM treatment in 2009 [50]. It can be administered as monotherapy or in combination with TMZ for enhanced anti-cancer effects. Recent studies have investigated the conjugation of bevacizumab and other VEGF-targeting antibodies to nanoparticles to improve drug delivery and tumor- targeting properties. For example, VEGF-targeting antibodies have been conjugated to liposomal nanoparticles in preclinical models, such as tumor-bearing rats with C6 glioma. This strategy significantly enhanced cellular uptake of the encapsulated anti-cancer drug, suggesting improved therapeutic efficacy and specificity [51]. Antibodies can also be designed to target specific genetic alterations in cancer cells. One such target is the p53 RH175H mutant, a common mutation that impairs the tumor suppressive function of TP53. Antibodies developed to recognize this mutation can help restore p53 activity, thereby inducing cancer cells apoptosis [52].

### Emerging antibody strategies

Building on the established utility of monoclonal antibodies in targeting tumor-specific markers, emerging strategies have focused on enhancing their cytotoxic potential through conjugation with potent chemotherapeutic agents. Monoclonal antibodies (mAbs) have been successfully employed in the treatment of a wide range of cancers, making them a versatile treatment strategy. mAbs targeting GBM-specific or overexpressed antigens, particularly in the form of antibody-drug conjugates (ADCs), have shown promising potential in preclinical and early phase clinical studies [53]. ADCs are a class of targeted therapies that combine the specificity of mAbs with the cytotoxicity of chemotherapeutic agents. Several ADCs have received FDA approval in recent years for non-CNA cancers, supporting their potential utility in brain tumor therapy [54].

One such example is mirvetuximab soravtansine, an ADC targeting the folate receptor alpha (FRα), which is overexpressed in ovarian cancer. This drug delivers a maytansinoid payload that disrupts microtubule formation by inhibiting tubulin polymerizsation, ultimately leading to mitotic arrest and apoptosis [55]. Another ADC, tisotumab vedotin (commercially known as Tivdak) targets tissue factor and was approved in 2021 for the treatment of cervical cancer. In a clinical trial, it showed a 24% overall response rate, including complete and partial responses. However, patients experienced adverse effects ranging from mild (alopecia, nausea), to more severe neutropenia [56]. Traztuzumab deruxtecan, an anti-HER2 ADC have demonstrated significant efficacy in low expressing breast cancer and was approved in 2022. Unlike earlier HER2-targeted therapies that primarily benefit patients with high HER2 expression, this ADC showed broader applicability and greater therapeutic efficacy [57]. It works by inhibiting topoisomerase I, stabilizing the cleavable complex between the enzyme and DNA during replication and transcription, which results in DNA damage and cancer cell death [58]. Despite its clinical benefit, grade 3 adverse events were reported in 52.6% of patients [57].

Aidixi, also known as disitamab vedotin, is another HER2-targeting ADC developed for monomethyl auristatin E (MMAE), which disrupts microtubule organization and impairs G2 or M phase, inducing apoptosis. Although generally well-tolerated, off-target effects, such as neutropenia, leukopenia, and even some treatment-related deaths were reported, reflecting the potential risks associated with ADCs [59].

While no ADCs have yet received FDA approval specifically for brain tumors, many pre-clinical and clinical studies are being conducted following the success of ADCs in other cancers. A notable example is AGCM-22, a novel ADC combining cetuximab (anti-EGFR mAb) with MMAE. In vitro studies demonstrated that AGCM-22 inhibits GBM cell proliferation via apoptosis, and in vivo experiments showed enhanced tumor targeting and significant suppression of GBM growth when compared to TMZ. These findings suggest that cetuximab-based ADCs may provide a targeted to treat EGFR-overexpressing GBM cells [60].

Another ADC targeting EGFR-expressing brain tumors is depatuxizumab mafodotin. While this ADC employs similar targeting mechanism as cetuximab-based therapies, its efficacy in GBM has been limited due to insufficient drug delivery across BBB [61]. A recent Phase III trial failed to demonstrate a survival advantage in GBM patients treated with depatuxizumab mafodotin. While progression-free survival was significantly higher in ADC group compared to controls, no significant improvement in overall survival (OS) was observed—with median OS being 18.9 vs. 18.7 months, respectively. These findings underscore the critical need for alternative and more effective drug delivery systems to enhance ADC efficacy in brain cancer [62].

Moreover, bispecific and trispecific antibodies have emerged as promising treatment candidates, offering the advantage of engaging multiple tumor-associated antigens and immune effector cells simultaneously. In pre-clinical GBM models, these engineered antibodies have demonstrated strong immune cell activation and enhanced anti-tumor efficacy [63]. Daniel et al. developed tri-specific T-cell engagers targeting EGFRvIII and IL-13Rα2 (tumor-associated antigens), along with CD3 on T cells, achieved complete tumor eradication in a heterogeneous intracranial GBM mouse model. This approach significantly outperformed monospecific antibody controls, achieving a 67% survival rate in vivo. Importantly, this trispecific antibody was designed to overcome the challenges of tumor heterogeneity, especially in GBM cases lacking uniform antigen expression [64].

Similarly, bispecific T-cell engagers (BiTE) are being investigated in early-phase trials [65]. For instance, AMG596, a BiTE targeting EGFRvIII and CD3, was evaluated in patients with current GBM or malignant glioma. In this study, one out of eight patients experienced partial response and two had stable disease, with no treatment-related toxicity observed. However, 50% of the patients reported adverse side effects such as headaches, and pharmacokinetic limitations remain a challenge—particularly related to administration routes (NCT number: NCT03296696).

### Limitations of current treatments

Despite significant advancements in surgical resection, radiotherapy, and chemotherapy, the prognosis for brain cancer, particularly glioblastoma, remains poor. This is largely due to the invasive nature of the disease, treatment resistance, and the unique anatomical and physiological challenges posed by the CNS [66]. Surgical resection, although a mainstay in treatment, carries substantial risks [67]. Incomplete tumor removal is common due to the infiltrative nature of GBM, and procedures are often associated with complications such as infection, hemorrhage, and postoperative cerebral edema, which can lead to serious complications including stroke [24]. While corticosteroids are often administered to reduce swelling, patients may still experience transient symptoms such as headaches, dizziness, poor coordination and blurred vision. Long-term surgical complications are often more serious, particularly when tumors are located near or within eloquent brain regions. These may include motor dysfunction, cognitive impairments, language deficits, and seizures [68]. For instance, resection involving the frontal lobe, paracentral lobule, or corticospinal tract often leads to impaired mobility and fatigue [69]. Inadvertent damage to temporal or parietal lobes may result in memory loss, visuospatial disorientation, or language difficulties [70]. The type, size, and location of the tumor, along with the surgical approach, greatly influence the extent and type of neurological deficit experienced [71].

Radiotherapy, while an important adjunct or alternative to surgery, is also associated with off-target effects due to its limited selectivity for tumor tissue. Even with precision technologies such as intensity-modulated radiation therapy (IMRT) or stereotactic radiosurgery, adjacent healthy tissues may be inadvertently damaged. This can result in alopecia, radiation necrosis, vascular damage, leading to long-term complications such as cognitive decline and sensory deficits [27, 72]. Cognitive impairments are well-documented in patients receiving cranial irradiation, particularly whole-brain radiotherapy (WBRT). Months or years post-treatment, patients may experience memory loss, mental confusion, or difficulty concentrating [73]. Additionally, sensory complications such as partial vision loss, blurred vision, or cataracts (from optic nerve damage), and hearing loss (from irradiation of auditory pathways) are often underreported but significantly impact quality of life [72].

Another key challenge across all treatment modalities is the BBB. This selective barrier severely restricts the entry of most chemotherapeutic agents—especially those with molecular weights above 500 Da or lacking sufficient lipophilicity—into the brain parenchyma, thus limiting drug accumulation at the tumor site and reducing therapeutic efficacy [74]. Many conventional chemotherapeutic agents, including doxorubicin (DOX) and paclitaxel, exhibit limited brain penetration due to poor BBB permeability and are rapidly eliminated from the CNS. Moreover, these agents are often pumped back into circulation via efflux transporters, such as P-glycoprotein (P-gp) and breast cancer resistance protein (BCRP) [75]. Additional challenges associated with these agents include poor pharmacokinetics, drug resistance, and low tumor specificity. For instance, TMZ exhibits poor BBB penetration, limited stability, and short half-life. Consequently, patients are often administered higher doses, which often results in systemic toxicity. TMZ resistance is also increasingly reported, reducing its anti-tumor activity [76].

Similarly, clinical trials investigating tandem regimens such as tandutinib (a tyrosine kinase inhibitor) with bevacizumab (a VEGF inhibitor) have shown no clinical benefit [25]. A Phase II study found that the combination failed to improve overall response or survival rates compared to bevacizumab monotherapy in patients with recurrent GBM [77].

While immunotherapy has demonstrated promising therapeutic outcomes across various cancer types, its effectiveness in GBM has been disappointing, in the later stage trials [78]. The highly immunosuppressive tumor microenvironment, low neoantigen burden, and restricted drug delivery across the BBB classify GBM as a ‘cold’ tumor, which is poorly responsive to immune checkpoint blockade [41, 78]. In several Phase III trials, immune checkpoint inhibitors (ICIs) such as nivolumab (anti-PD1 checkpoint inhibitor) failed to demonstrate significant increase in median overall survival or median progression-free survival when administered with other anti-tumor drugs in GBM patients [79, 80]. Likewise, pembrolizumab, approved for various cancer types including breast, head and neck cancer, has shown no substantial benefit in GBM. For example, a Phase I study evaluating its combination with CAR T-cell therapy showed no significant improvement in median overall survival (NCT number: NCT03726515).

Although monoclonal antibodies offer superior specificity, clinical outcomes remain inconsistent due to several limitations [81]. For example, bevacizumab, has not demonstrated significant improvement in overall survival in brain cancer patients, and tumor relapses are common. Furthermore, adverse effects such as hypertension and proteinuria have raised concerns about its safety [82]. Consequently, bevacizumab is not approved for brain tumor treatment in the UK and Europe, despite its FDA approval in the U.S [83].

Trastuzumab, another monoclonal antibody (mAb) approved for HER2-positive brain metastases from breast cancer, shows limited efficacy. While it may symptom progression, it is ineffective in stopping brain tumor progression [84]. One of the critical challenges for all mAbs is their inability to effectively cross the BBB. Owing to their molecular size of about 150 kDa and hydrophilicity, less than 0.1% of systemically administered antibodies reach the brain parenchyma, resulting in subtherapeutic concentration at the tumor site [85].

To overcome these limitations, antibody-conjugated nanoparticle-based drug delivery systems have emerged as a promising strategy. These nano-systems are designed to enhance drug targeting, limit off-target effects, and enhance BBB penetration [39]. For example, loading of TMZ into polymer nanoparticles has been shown to improve its stability and solubility, reduce systemic toxicity, and increase drug accumulation in brain tissue, thereby enhancing therapeutic efficacy [86].

## BBB (BBB) as a biological barrier

**Fig. 1:**
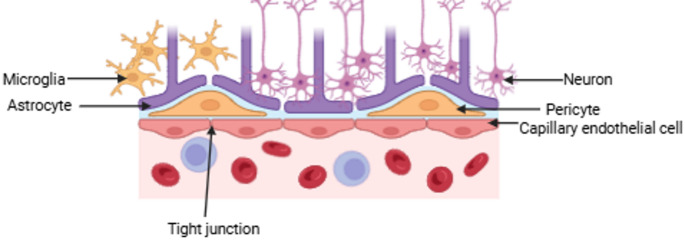
Structure of the BBB. Tight junctions between endothelial cells in the BBB (BBB) form a dense network that prevents substances from passing through. These tightly sealed junctions leave no gaps, creating a formidable barrier to the entry of most drugs, which are unable to penetrate the brain tissue due to this highly selective and protective mechanism. As a result, therapeutic agents face significant challenges in crossing the BBB, limiting the effectiveness of many treatments for brain-related conditions and diseases, including brain cancer. Glial cells, such as astrocytes and microglia, are also shown in the diagram

The BBB serves as a highly selective physiological interface that protects the CNS by regulating the passage of substances between the bloodstream and the brain [87]. The BBB is composed by microvascular endothelial cells connected by tight and adherent junctions, which include transmembrane proteins such as claudins. These structural components maintain a controlled microenvironment in the brain interstitial fluid by maintaining optimal ionic composition and preventing the entry of exogenous or potentially harmful compounds [87, 88]. As shown in Fig. 1, the BBB is made of microvascular endothelial cells lining the cerebral capillaries that supply blood to the brain and spinal cord. Its high selectivity prevents most macromolecules and hydrophilic substances crossing, unless transport is mediated by specific receptors expressed on the endothelial cells. Only small, lipophilic molecules with a molecular weight of less than 400 Da can cross the BBB via passive diffusion [89]. Studies have shown that approximately 98% of small molecules are excluded by the BBB, limiting the utility of many chemotherapeutic agents [13]. Although increasing a drug’s lipophilicity can enhance BBB penetration, this often comes at the cost of reduced therapeutic specificity, increased off-target interactions, and prolonged systemic exposure [13]. In addition, the BBB expresses ATP-binding cassette (ABC) transporters, such as P-glycoprotein (P-gp) and breast cancer resistance protein (BCRP), which actively efflux drugs back into the bloodstream, further reducing intracerebral drug accumulation and contributing to therapeutic resistance [90].

Various current delivery strategies have been employed to circumvent the BBB such as intrathecal administration or BBB disruption with intracranial implantation, convection-enhanced delivery (CED) and deep-brain stimulation methods. However, these approaches are invasive and may result in serious complications like cerebral inflammation and neurotoxicitiy [13]. In vivo investigations have reported excessive toxicity with DOX administered via CED to the pons [91], and a Phase I/II study conducted by Lidar et al. reveal complications such as meningitis and extravasation in patients undergoing CED-based chemotherapy [92]. Non-invasive methods such as focused ultrasound (FUS) have been explored to transiently open the BBB and enhance drug permeability [93]. While FUS has shown promise in enhancing BBB permeability, its lack of selectivity poses significant risk, allowing both therapeutic and neurotoxic agents to enter the brain parenchyma. Prolonged or high-intensity ultrasound exposure can result in brain oedema, tissue damage and neuronal cell death, necessitating cautious selection of treatment parameters [94].

## Nanoparticles as a drug delivery vehicle

To address the challenge of crossing the BBB, nanoparticles have emerged as a promising solution for delivering drugs directly to the brain. Nanoparticles are typically 1–100 nm in size; and are classified based on their structural and chemical properties [95]. Various types of nanoparticles have been explored for treating brain cancer in preclinical and clinical studies. These include polymer nanoparticles, solid lipid nanoparticles, inorganic metal-based nanoparticles, and carbon-based nanoparticles [96]. Typically ranging from 1 to 100 nm in size, nanoparticles can be generally classified in a broad context based on their material composition. By virtue of their small sizes, nanoparticles can easily be transported across numerous sites and possess an enormous surface area to volume ratio [97]. Apart from the direct benefits granted by their small sizes, nanoparticles can be readily modified through various means to afford selective targeting, controlled drug release, stimuli-responsive behavior, and other advanced functionalities. These key attributes have positioned nanoparticles as a major focus of research in the broader field of drug delivery [95, 98].

In the context of brain-targeted drug delivery, common types of nanoparticles include polymeric nanoparticles, lipid-based nanoparticles, nanovesicles, and inorganic metal-based nanoparticles [99, 100]. Moreover, as the BBB endothelial cells are rich in negatively charged molecules like proteoglycans and glycosaminoglycans (GAGs), nanoparticles with a slight positive or neutral surface charge exhibit enhanced interaction and permeability across the barrier [98]. Finally, similar to the transport of larger endogenous molecules, nanoparticles can cross the BBB via receptor-mediated transcytosis [15] This process involves specific transporter or carrier proteins expressed on the endothelial surface. For instance, insulin and transferrin receptors, both highly expressed in BBB endothelial cells, can facilitate nanoparticle uptake when their respective ligands are conjugated to the nanoparticle surface, allowing internalization of the therapeutic cargo across the BBB [101, 102].

## Polymer nanoparticles as a drug delivery vehicle

**Fig. 2:**
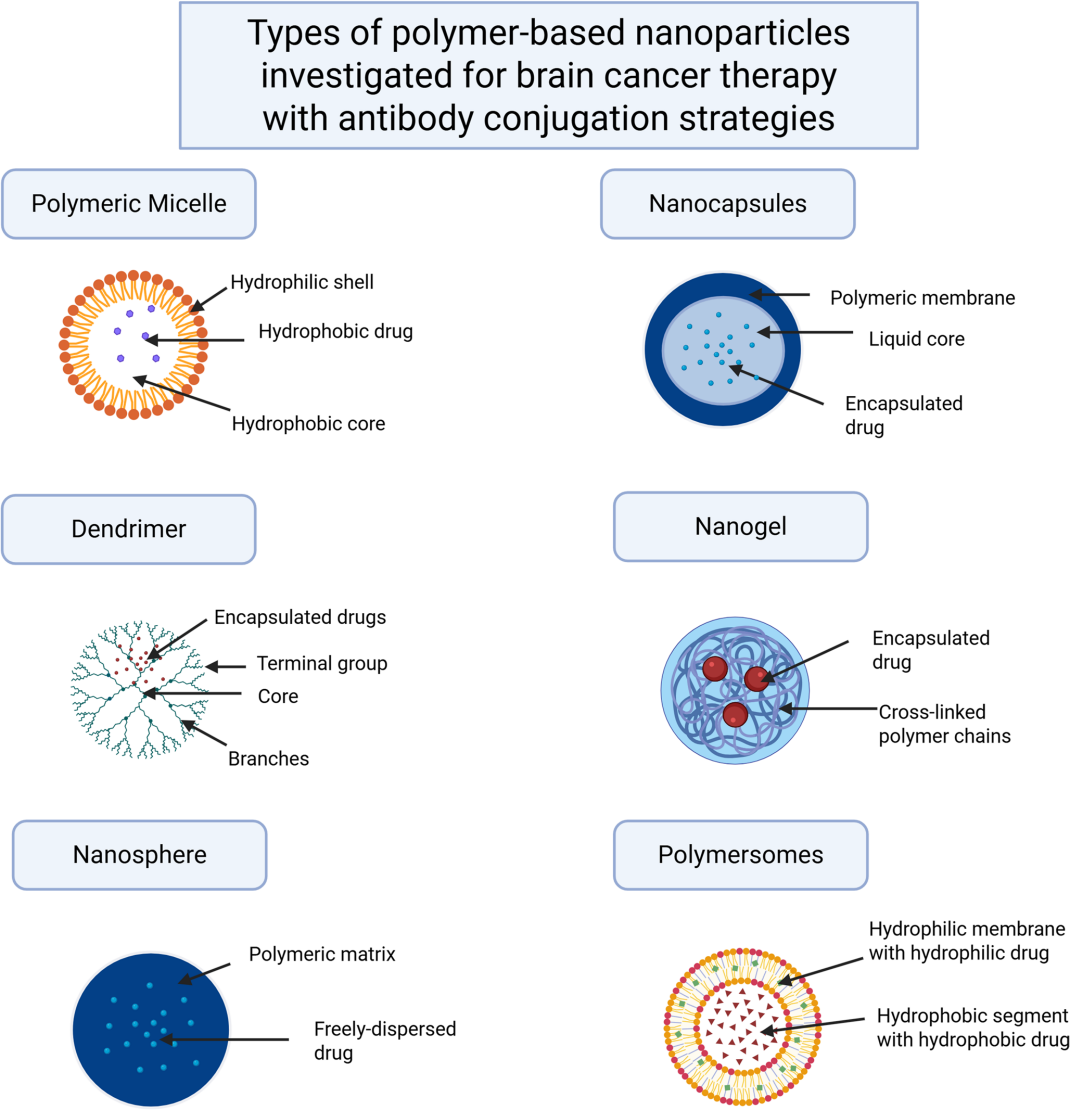
Types of polymer nanoparticles used as drug delivery vehicles for brain cancer therapy. Image was generated by Biorender

Polymeric nanoparticles have been extensively investigated as drug delivery vehicles in the context of brain cancer. These include various distinct classes such as polymeric micelles, nanocapsules, nanospheres, dendrimers, nanogels, and polymersomes, as illustrated in Fig. 2 [103]. A key advantage of polymeric nanoparticles is their enhanced tumor selectivity, which minimizes off-target effects and systemic toxicity [104]. Through extensive research spanning decades, polymeric nanoparticles have evidently possessed capabilities of reducing cytotoxicity towards healthy non-cancerous cells, increase drug permeation across the BBB, enhance antitumor efficacy, prolong drug half-life, and improve overall bioavailability. These features allow polymeric nanoparticles to overcome key limitations of conventional chemotherapy, such as poor drug solubility, rapid drug clearance from the circulation, and low tolerability as a direct consequence of nonspecific toxicity due to low selectivity for cancer cells [104–106].

The inherent biodegradability and biocompatibility of polymeric nanoparticles based on the choice of polymers used, also makes them a good candidate as they reduce the risk of eliciting toxic immunological responses by the host immune system [107]. Moreover, their ability to attain sustained drug release potentially enables the reduction of dosing frequency, improving patient compliance and acceptability [108]. Polymeric nanoparticles also allow for the co-delivery of therapeutic compounds alongside other agents from the same class or different nature such as chemotherapeutic drugs and immunological agents [109].

Polymeric nanoparticles also possess the inherent advantage of having easily modifiable surfaces, directly allowing for diverse range of augmentations. To further improve circulation time and targeting efficiency, a common strategy employed in the field of drug delivery is surface modification through PEGylation. By doing so, the modified polymeric nanoparticles can evade clearance by phagocytes, allowing the nanoparticles and their therapeutic cargo to remain active for a longer duration [110]. For instance, a study had demonstrated that polymeric micelles loaded with TMZ achieved sustained drug release in the brain and significantly improved antitumor activity in U87MG tumor-bearing nude mice [109]. Similarly, a separate study by Sharma et al. revealed a higher cellular uptake of the drug TMZ by glioma cancer cells when delivered using a polyamidoamine (PAMAM) dendrimer while extending the drug’s half-life by 1.5-fold compared to the free drug [111].

One of the most widely reported polymers used in nanoparticle-based drug delivery is poly (lactic-co-glycolic acid) (PLGA) – a negatively charged amphiphilic copolymer composed of poly(glycolic acid) (PGA) and polylactic acid) (PLA) [112]. Maksimenko et al. utilized PLGA to encapsulate drugs DOX observing a biphasic release profile. Approximately 70% of encapsulated DOX was released within 6 h when the PLGA-based nanoparticle was suspended in water, in contrast to only 40% being released in PBS over the same period [113]. Poly(caprolactone) (PCL), another well-known polymer, is synthesized from the hydrophobic monomer ε-caprolactone [114]. Due to its biodegradability and low toxicity, PCL is regarded as one of the ideal candidates for biomedical applications. PCL-based nanoparticle systems are able to offer unique properties tailored to specific applications such as tunable degradation rate and solubility, by blending it with other polymers [114]. PAMAM, commonly used in dendrimer-based systems, represents another commonly used polymer for drug delivery [115]. While PAMAM exhibits inherent cytotoxicity at high concentrations, surface modifications can circumvent this limitation, significantly reducing its toxicity towards healthy cells. Conjugation with molecules such as phenylalanine, histidine, or arginine with PAMAM not only improves biocompatibility but also enhances targeted delivery of the therapeutic cargo [115]. For instance, PAMAM dendrimers modified with arginine have demonstrated enhanced gene transfection efficiency, significantly improving apoptin gene delivery to glioma cells compared to unmodified PAMAM [115, 116]. In addition to synthetic polymers, naturally derived polymers are also commonly used as a building material for polymeric nanoparticles [117]. Chitosan, obtained through the deacetylation of chitin, is among the most frequently utilized natural polymers for targeting glioblastoma, owing to its excellent biodegradability and safety profile. Notably, the inherent therapeutic properties of chitosan, including antibacterial, antifungal, antitumor, and neuroprotection, make it a highly versatile candidate for brain-targeted drug delivery [117].

### Active targeting mechanism

**Fig. 3:**
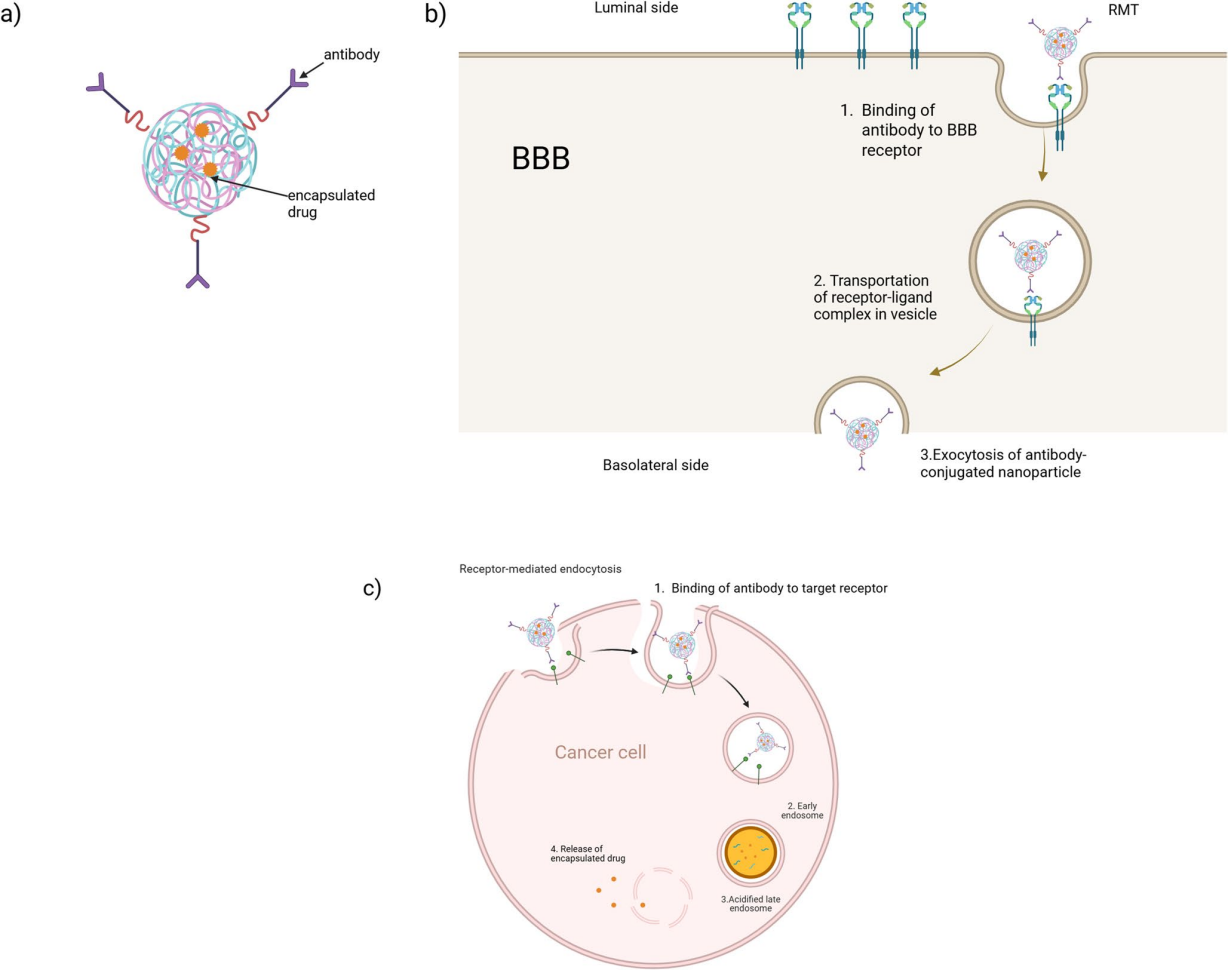
Interaction of antibody on polymer nanoparticle surface with receptors expressed on the BBB endothelium for uptake of nanoparticles. Images were generated by Biorender. Figure 3a Antibody-conjugated polymer nanoparticle with drug encapsulated in matrix. Antibodies can be conjugated on the surface of a nanoparticle via various chemical interactions like covalent bond formation. Figure 3b Active-targeting mechanism of antibody-conjugated polymer nanoparticles. Nanoparticles can cross the BBB via interactions with specific surface receptors on BBB endothelial cells to reach tumor cells followed by receptor-mediated transcytosis (RMT). Figure 3c Antibodies can also interact with tumor cell receptors, allowing specific targeting. The figure also demonstrates the mechanisms of receptor-mediated endocytosis

Ligand-mediated targeting is an advanced nanotechnological strategy aimed at enhancing the specificity and therapeutic efficacy of nanoparticle-based drug delivery systems for brain cancer [118]. This approach involves the surface functionalization of nanoparticles with targeting ligands—such as peptides, antibodies, aptamers, or small molecules—that selectively bind to overexpressed receptors on tumor cells or within the tumor microenvironment. Known as active targeting, this strategy relies on clathrin-mediated endocytosis and facilitates not only the accumulation of therapeutic agents at the tumor site but also aids in overcoming physiological barriers such as the blood–brain barrier (BBB) and in improving cellular uptake (Fig. 3) [118, 119].

A major advancement in this area is the conjugation of monoclonal antibodies or antibody fragments onto nanoparticle surfaces, enabling selective recognition and binding to specific receptors expressed on BBB endothelial cells [120]. This technique exploits receptor-mediated transcytosis (RMT)—a natural transport mechanism that allows for the internalization and translocation of macromolecules across the BBB [120]. By leveraging this pathway via antibody-conjugated polymer nanoparticles, drug availability at the target site can be enhanced, and systemic toxicity can be minimized [118, 119]. Commonly exploited ligands include folate, transferrin, epidermal growth factor (EGF), integrins (e.g., αvβ3), and interleukin receptors, with the most notable one being antibodies [121].

Upon binding to its target receptor on the luminal surface of the BBB endothelium, the newly formed ligand–receptor complex is internalized via clathrin-mediated endocytosis, the primary mechanism of RMT. This process begins with the invagination of the plasma membrane to form clathrin-coated pits, which then pinch off to form intracellular vesicles. After uncoating, these vesicles fuse with early endosomes, from which the receptor–ligand complex can be sorted and trafficked to the basolateral membrane for exocytosis into the brain parenchyma, as illustrated in Fig. 3 [15].

### Interaction between nanoparticles and brain cancer cells

Antibody-conjugated nanoparticles enable the selective targeting of brain cancer cells by exploiting surface receptors that are overexpressed both on tumor cells and on the endothelial cells of the blood–brain barrier (BBB), such as transferrin receptor (TfR) and epidermal growth factor receptor (EGFR) [1]. Upon binding to the target receptor, these nanoconjugates are internalized via receptor-mediated endocytosis, most commonly through clathrin-mediated pathways—a mechanism closely resembling receptor-mediated transcytosis (RMT) across the BBB [122].

Once internalized, the therapeutic payload is typically released through the endosomal–lysosomal pathway [123]. As the endocytic vesicles mature from early endosomes into late endosomes and ultimately lysosomes, the intracellular environment becomes increasingly acidic. This drop in pH, combined with enzymatic activity, facilitates the cleavage of pH-sensitive linkers, including hydrazone, acetal, and cis-aconityl, and enzymatically cleavable linkers, triggering drug release [123, 124]. These chemically engineered linkers ensure that drug release occurs specifically within the intracellular compartments of target cells, thereby minimizing premature release at off-target sites [123].

### Target ligands/ receptors/ transporters

#### Tyrosine kinase receptors

The tyrosine kinase receptor is highly expressed in the BBB and glioma cells. It is responsible for signaling cascades that contribute to tumor invasion and angiogenesis in brain cancer. EGFR is a well-known example of a tyrosine kinase receptor expressed in abundance on both the BBB and glioma cells [124]. Monoclonal antibodies targeting EGFR, such as cetuximab and panitumumab have promising clinical outcomes, demonstrating improved tumor inhibition and increased survival rates in glioblastoma patients. Consequently, conjugating anti-EGFR antibodies to nanoparticles represents a viable strategy to enhance targeted delivery and therapeutic efficacy in glioblastoma treatment [124].

In recent years, research surrounding the use of EGFR-targeting antibody-functionalized polymeric nanoparticles have gained much interest. Beyond in vitro studies, therapeutic benefits have also been demonstrated in vivo. For example, in a glioma rat model, dendrimers conjugated with anti-EGFR antibodies significantly improved survival when used in combination with boron neutron capture therapy (BNCT), with treated rats showing a 107% increase in lifespan compared to controls [125].

However, certain limitations persist whereby specific variants of EGFR such as wild-type EGFR is also widely expressed in non-brain tissues (skin, lungs, and gut), posing a risk of off-target effects [126]. This underscores the importance of selecting tumor-specific EGFR variants, or alternative brain tumor-specific receptors, and employing targeted delivery systems like antibody-functionalized nanoparticles. Such strategies will be critical to improving selective delivery to the tumor site while minimizing unwanted adverse effects [121].

#### Transferrin receptors

Monoclonal antibodies can be engineered to target transferrin receptors (TfR), which are abundantly expressed on both the endothelial cells of the blood–brain barrier (BBB) and on various brain cancer cells. TfR plays a critical physiological role in maintaining iron homeostasis by mediating the uptake of transferrin-bound iron via receptor-mediated endocytosis [127]. A well-studied example is the monoclonal antibody OX26, which specifically binds to the CD71 isoform of TfR. When conjugated to polymeric micelles, OX26 significantly enhanced nanoparticle uptake by C6 glioma cells, demonstrated through flow cytometry [128].

In recent studies, alternative TfR-targeting molecules, such as the nanobody M1, have been explored as pH-dependent shuttle systems for transcytosis. These constructs demonstrate favourable binding at physiological pH while releasing the therapeutic cargo under acidic endosomal conditions following endocytosis [127]. This mechanism facilitates effective drug delivery across the BBB. Favourable pharmacokinetic profiles have been observed, with nanomolar concentrations detected in capillary-depleted brain lysates following intravenous administration of the intervention. However, the current M1 construct is specific only to murine TfR, highlighting the need for further molecular engineering to generate human-compatible versions suitable for clinical applications [127].

It is also important to consider that the efficiency of antibody-mediated TfR targeting depends on both receptor density and nanoparticle size. For instance, antibody-conjugated particles exceeding 5 μm in diameter typically exhibit poor internalization by target cells [129]. Moreover, while M1 nanobodies show promise in preclinical models, their utility in therapeutic or diagnostic imaging applications remains to be fully validated, and comprehensive safety evaluations are still required to support clinical translation [130].

#### Glucose transporter 1 (GLUT1)

By and large, GLUT1 is also frequently overexpressed in various gliomas and other CNS tumors due to their elevated metabolic demands [131]. This overexpression makes GLUT1 a promising target for brain-directed nanocarrier systems. Early studies aimed to enhance drug delivery across the BBB by conjugating multiple glucose moieties onto the surface of nanoparticles, thereby increasing their affinity for GLUT1 and enabling transport via GLUT1-mediated transcytosis [132]. For example, Anrku et al. developed PEG-based polymeric micelles functionalized with glucose and observed a 20-fold increase in nanocarrier uptake in a glioma murine model [133]. Consequently, several recent studies have demonstrated the feasibility of employing GLUT1-specific ligands, including antibodies and single-chain variable fragments (scFvs), to improve nanoparticle delivery to brain tumors [134]. In a notable example, Abouzeid et al. developed anti-GLUT-1 antibody-decorated PEG-PE micelles co-encapsulating both curcumin and DOX for targeting HCT-116 cells [135]. These antibody-conjugated micelles significantly improved both in vitro and in vivo tumor inhibition in HCT-116 cell models and extended survival rates in treated animals. In contrast, non-targeted micelles showed no significant therapeutic benefit compared to untreated controls. These findings highlight the potential of GLUT1-targeting antibody-nanoparticle formulations in improving tumor specificity and inhibition. Further optimization of these systems, focusing on controlled release, immune compatibility, and stability, could pave the way for more effective translational applications [136].

#### Insulin receptors

Insulin receptors, which are abundantly expressed on the BBB, represent another promising target for receptor-mediated transcytosis. This transport mechanism is essential for delivering insulin into the brain, where it is needed to support metabolic regulation and cognitive function [137]. Disruption of this signaling pathway has been implicated in the CNS, leading to insulin resistance, a pathological feature associated with several neurodegenerative disorders, including Alzheimer’s disease, Parkinson’s disease, and Huntington’s disease [138]. Targeting the insulin receptor has been explored as a strategy to enhance nanoparticle-mediated drug delivery to the brain. For instance, in 2013, Kuo and Ko developed solid lipid nanoparticles conjugated with the 83 − 14 monoclonal antibody, which specifically binds to the insulin receptor, to deliver Saquinavir, an antiretroviral agent, to the CNS [139]. Their study demonstrated that antibody-functionalized nanoparticles exhibited significantly increased cellular uptake and improved drug bioavailability, with uptake levels positively correlating with antibody concentration. While this study focused on the application of the nanoformulation for AIDS therapy, the efficient BBB penetration demonstrated by the nanocarrier holds significant promise for broader applications in CNS diseases, including brain cancer [139].

### Programmed death 1 (PD1)

Antibody conjugation strategies can also be utilized to combine chemotherapy and immunotherapy within a single nanoparticle system. Zhan et al. attached anti-PD1 antibodies to polymeric micelles loaded with DOX [46]. Anti-PD1-antibodies block the interactions between the PD-1 receptor expressed on activated T-cells and its ligands commonly overexpressed on cancer cells. This interaction inhibits T cell activity, preventing immune cells from recognizing tumor cells. By inhibiting the interactions, anti-PD1 antibodies promote the activation of cytotoxic T cells, promoting immune responses against cancer cells [140]. Notably, these polymeric micelles were designed to release DOX more rapidly under acidic conditions, such as those found in the tumor microenvironment, resulting in enhanced drug accumulation at the target site [46]. The antibody-functionalized polymeric micelles demonstrated significantly delayed tumor growth in vitro, accompanied by increased recruitment ok NK cells and T-cells compared to the unmodified polymeric micelles. Additionally, this formulation exhibited a substantially improved safety profile relative to free DOX [46]. In a related in vivo study, administration of anti-PD-1 antibody-conjugated polymeric micelles in a murine tumor model led to heightened immune responses and increased rates of apoptosis within tumor tissues [141].

## Antibody-conjugation for ligand-directed delivery of polymer nanoparticles

### Method of conjugation

Polymer nanoparticles can be functionalized using a variety of conjugation methods, each offering distinct benefits depending on the intended application. The choice of method can influence antibody orientation, stability, and biological activity, which altogether affect the overall therapeutic performance of the drug delivery system [142]. While some techniques enable efficient tumor targeting recognition via facilitating site-specific and high-affinity antibody conjugation to nanoparticle surfaces, this often comes at a higher cost of time and money [143]. Thus, various factors such as cost, desired release profile, and drug delivery applications need be taken into consideration when selecting the conjugation strategy.

### Adsorption

Functionalizing polymeric nanoparticles with antibodies requires careful consideration of factors such as antibody orientation, surface density, and binding strength to achieve optimal biological activity and targeting efficiency [144]. One commonly used method is adsorption, in which antibodies are non-covalently attached to the nanoparticle surface via electrostatic interactions, van der Waals forces, or hydrophobic interactions [12]. This approach does not require prior chemical modification of the antibodies, making it relatively simple and versatile. However, the inherently weaker and reversible nature of these interactions results in lower stability compared to covalent bonding [145]. Nevertheless, the transient nature of adsorption-based conjugation can offer advantages in specific contexts, such as facilitating the controlled release of antibodies or enhancing tumor targeting by enabling localized and timely presentation of antibody-mediated anti-tumor activity at the disease site [146].

### Carbodiimide chemistry

In contrast, covalent conjugation strategies are more widely employed due to their superior stability and prolonged retention of antibodies on the nanoparticle surface. These methods typically involve the activation of specific functional groups, such as carboxyl, hydroxyl, or amine moieties of the polymer structure on the nanoparticle surface, to enable the formation of stable covalent bonds [144]. A commonly used approach is carbodiimide chemistry, wherein a primary amine group on the antibody reacts with an activated carboxyl group on the polymer chain to form a stable amide linkage [147]. Despite its effectiveness, this method can result in random antibody orientation and undesired cross-linking, as antibodies often possess multiple reactive amine sites. Such variability can hinder reproducibility and standardization in nanoparticle production, potentially jeopardizing targeting efficiency and therapeutic performance [148]. An example can be seen in a study where anti-GD2 3F8 mAb were conjugated to PLGA and PLA polymer nanoparticles via EDC/NHS-mediated amide bond formation between the primary amine groups on the antibody and the carboxyl groups on the polymer chain [149].

### Meleimide-thiol chemistry

Another widely used covalent conjugation strategy is maleimide-thiol chemistry, which exploits the high reactivity between maleimide groups on polymer chains and sulfhydryl (–SH) groups on antibodies [150]. These linkages are typically introduced through the deprotonation of native sulfhydryl or via site-specific engineering of cysteine residues. The resulting thioether bonds are highly stable and form more rapidly and selectively than amide bonds in carbodiimide-mediated reactions, owing to the greater affinity of maleimide for thiol groups compared to primary amines [151]. This approach offers enhanced specificity and controlled antibody orientation. However, maleimide-thiol conjugation often requires pre-modification of the antibody to introduce free sulfhydryl groups at defined sites. These additional steps can be labor intensive, time-consuming, and costly, thereby limiting its scalability for clinical applications [146]. In a study by Yue et al., OX26 antibody was thiolated using Traut’s reagent prior to its conjugation onto the surface of MRhB hybrid micelles in a thermostatted oscillator set [152].

### Click chemistry

Click chemistry has emerged as a widely used method for antibody-nanoparticle conjugation due to its high specificity, efficiency, and biorthogonality [153]. This approach typically involves the coupling of azide- and alkyne- functionalized groups on either the antibody or polymer chain. The copper (I)-catalyzed azide-alkyne cycloaddition (CuAAC) is a well-established click reaction that has been extensively used in peptide and polymer conjugation [154]. However, the use of copper catalysts poses a cytotoxicity risks, limiting its applicability in biomedicine [155]. To address this limitation, copper-free click chemistry methods, such as strain-promoted azide-alkyne cycloaddition (SPAAC), has gained popularity. SPAAC utilizes strained cyclooctyne derivatives, such as dibenzocyclooctyne (DBCO), which react rapidly and selectively with azide groups under physiological conditions, thereby eliminating the need for toxic metal catalysts while preserving high reaction efficiency [156]. A notable example can be found in a study by Yang et al. where CD163-conjugated polymer nanoparticles were synthesized using SPAAC. Each mAb was functionalized with an average of 3.1 DBCO molecules before being conjugation to the azide-modified nanoparticles [157]. Despite its advantages, click chemistry requires pre-functionalization of either the antibodies or nanoparticles, which results in a high cost and ultimately low output [146].

### Adapter molecules

To overcome the limitations of traditional covalent conjugation methods—such as random antibody orientation and potential loss of antigen-binding activity, adapter molecules can be employed in non-covalent conjugation strategies to achieve controlled and oriented immobilization of antibodies on nanoparticle surfaces [158]. This approach typically involves the specific attachment of the Fc region of the antibody, thereby preserving the antigen-binding Fab region for optimal interaction with target epitopes [158]. Among the most widely used systems in this context is the biotin–avidin interaction, which leverages the exceptionally high affinity between biotin and its binding proteins, such as avidin, a tetrameric glycoprotein [159].

This interaction forms a stable complex that remains intact under a broad pH and temperature range, making it suitable for robust bioconjugation [160]. However, due to the potential for non-specific binding associated with the glycosylation of avidin, non-glycosylated and neutrally charged avidin derivatives, such as streptavidin or neutravidin, are often preferred. Although these variants possess different pharmacological profiles than avidin, they offer enhanced specificity and reduced background interference [161].

### Characterization techniques

Diligence must be taken in measuring and determining the degree of antibody conjugation onto the surface of polymeric nanoparticles. Across the current literature, various techniques are available to researchers for this intention, providing valuable insight into conjugation efficiency. Additionally, such techniques allow for the quantification of antibody density and verification of surface immobilization. Such assessments are crucial for understanding the structural and functional aspects of these nanocarriers, which directly impact their biological performance and overall stability [162]. This section of the review highlights the key characterization techniques used measure and determine the conjugation of antibodies onto nanoparticles, with an emphasis on the unique roles provided by each method based on their underlying principles.

### Antibody-conjugation confirmation

#### Fourier-transform infrared (FTIR) spectroscopy

FTIR spectroscopy is a valuable analytical technique for examining chemical modifications on both polymer chains and by extension, polymeric nanoparticle surfaces, applicable to both solid and aqueous samples [162]. In the context of antibody conjugation, the formation of new covalent bonds such as amide, amine, or thioether linkages, can be detected through characteristic shifts in existing absorption peaks or the emergence of new peaks in the infrared spectrum. These spectral changes serve as indicators of successful conjugation and vary depending on the specific chemistry employed [162]. For example, in a recent study, the disappearance of a peak at 1743 cm⁻¹ indicated the loss of the carboxyl group on the PLGA nanoparticle surface, while the appearance peak at 1628 cm⁻¹ corresponded to the formation of an amide bond between PLGA and the antibody’s amine groups, confirming successful conjugation [163].

#### Proton nuclear magnetic resonance (1 H NMR)

Another useful spectroscopic technique is ^1^H NMR, offering unique advantages over FTIR, particularly in resolving a more detailed structure. In ^1^H NMR, peaks appear at specific chemical shift values based on the electron density surrounding each hydrogen atom [164]. Upon antibody conjugation, the emergence of new peaks or shifts in existing signals can indicate structural changes associated with bond formation. The simultaneous presence of characteristic peaks from both the antibody fragments and the polymer provides strong evidence of successful conjugation, confirming the incorporation of both components within the final nanoparticle construct [162]. ^1^H NMR was used in a study to confirm conjugation of anti-CD44v6 antibodies to the micelle made of amphiphilic polymer Pluronic^®^ F127. In the spectrum of the Fab-conjugated micelles (PM-NCS-Fab), two new characteristic signals appeared, including a peak at δ = 6.27 ppm, corresponding to olefinic protons from the maleic anhydride moiety, and a peak at δ = 2.54 ppm, attributed to aliphatic protons associated with the Fab-CD44v6 antibody, demonstrating effective functionalization of the micelles [165].

### X-ray photoelectron spectroscopy (XPS)

XPS is a highly surface-sensitive technique used to analyze the elemental composition and chemical states of materials within the top ~ 10 nm of the nanoparticle surface. In the context of antibody conjugation, the appearance of a distinct nitrogen (N) signal attributable with nitrogen-rich functional groups such as amines and peptide bonds present in antibodies, can serve as evidence of successful conjugation. This nitrogen peak is typically absent in non-functionalized nanoparticles, enabling clear differentiation between unmodified and modified nanoparticle surfaces [162]. Numerous studies have utilized XPS to confirm antibody attachment on polymeric nanoparticles, particularly in the development of targeted nanocarriers for cancer therapy [166–168].

#### Enzyme-linked immunosorbent assay (ELISA)

ELISA is a widely used technique for determining antibody conjugation and assessing the binding affinity of antibody-functionalized nanoformulations. In one study, it was demonstrated that GLUT-1 scFv-conjugated micelles exhibited specific binding to immobilized GLUT-1 protein, whereas no interaction was observed with non-targeted micelles or the IgG2a control [169]. These findings indicate the successful conjugation and preservation of the antibody’s targeting capability post-functionalization [169]. Similarly, indirect ELISA was employed in another study to verify the conjugation of anti-transferrin receptor (anti-TfR) and anti-2C5 antibodies to Polycefin nanoparticles. The assay demonstrated specific, dose-dependent binding of anti-2C5 to nucleosome-coated antigens and anti-TfR to transferrin receptor-coated antigens, further validating the functional integrity of the conjugated antibodies [170].

#### Sodium dodecyl sulfate–polyacrylamide gel electrophoresis (SDS-PAGE)

SDS-PAGE is an indirect yet informative technique capable of assessing antibody conjugation by separating proteins based on their molecular weight. In this technique, SDS, an anionic detergent, binds to proteins, imparting a uniform negative charge that enables size-dependent migration through a polyacrylamide gel under an electric field [171]. Upon staining, the banding patterns reflect the molecular size of the peptide chains. Antibody-conjugated nanoparticles typically produce bands corresponding to the molecular weights of the antibody’s heavy and light chains, or they may exhibit distinct band shifts compared to the free antibody due to the added mass of the nanoparticle [172]. In one study, a conjugate of PAMAM and mAb-CD133 exhibited a higher molecular weight band than mAb-CD133 alone, supporting successful conjugation [173]. Nevertheless, SDS-PAGE is not highly quantitative and may offer less definitive evidence compared to more robust techniques such as FTIR and ^1^H NMR [162].

### Targeting strategies involving mixed-ligand polymeric nanoparticles

Given the adaptable nature of polymers and the ease of modifying their surface chemistries, the integration of multiple antibodies into a single nanoparticle platform presents a compelling strategy to enhance therapeutic efficacy [127]. This strategy becomes additionally appealing upon realizing the diverse set of antibodies available to target the various ligands and receptors along the BBB and brain cancer cells [1, 120]. When designing such systems, antibody selection must be guided by the biological relevance of their respective targets [142]. Types of target receptors or ligands can be broadly grouped based on their relevance towards either the BBB or the tumor cells directly [100, 123]. Receptors such as transferrin, GLUT1, and insulin receptors, found highly expressed on the BBB, serve to facilitate transcytosis and promote nanoparticle accumulation in the brain [136, 154]. On the other hand, tumor-specific targets like tyrosine kinase receptors and PD-1 receptors, commonly overexpressed on glioma or glioblastoma cells, enhance selective uptake by malignant cells, minimizing off-target effects [126, 174]. By combining BBB-penetrating and tumor-targeting ligands in a single nanoparticle system, dual-targeting formulations capable of simultaneously improving brain delivery and intra-tumoral accumulation can be achieved [174]. However, incorporating multiple antibodies onto a single nanoparticle demands more complex surface engineering, introducing additional challenges in formulation design [148]. As nanotechnology continues to advance over the years, a small number of studies have since shown that such a premise could hold significant potential in treating brain cancer.

One such studies by Fujita et al. employed a dual-antibody polymeric nanoparticle system combining anti-mouse transferrin receptor antibody and a mouse autoimmune anti-nucleosome antibody C25. The poly(β-l-malic acid) nanoparticle system conjugated with this pair of antibodies carried a new Polycefin variant, in the form of antisense oligonucleotides to vascular protein laminin-8 as the antitumor drug component. Findings from this study revealed greater Polycefin accumulation in cultured glioma cells and through an in vivo athymic mice model (CrTac: NCr-Foxn1nu Homozygous, Taconic, USA), authors demonstrated improved tumor accumulation of Polycefin variants when the antibodies were used in combination compared to single antibodies [174].

Alternatively, the possible antibody combinations can be further expanded by incorporating immunotherapeutic antibodies with immune-modulating functions. Dominguez et al. functionalized PLA nanoparticles with anti-RNEU and anti-CD40 antibodies and studied the application of this system in producing enhanced anti-tumor activity. Authors from this study found that an antitumor response involving complete tumor regression and protective memory response generation was produced when using anti-neu/anti-CD40 nanoparticles but not anti-neu nanoparticles or anti-CD40 nanoparticles on their own. Formulated anti-neu/anti-CD40 nanoparticles also effectively targeted RNEU-positive tumor cells while simultaneously activating dendritic cells, highlighting their dual role in tumor recognition and immune stimulation [175].

## Types of antibody-conjugated polymer nanoparticles and their properties

The types of polymer nanoparticles (PNPs) discussed in this review include polymeric micelles, dendrimers, nanospheres, nanocapsules, nanogels, and polymersomes. Each of these nanocarriers possesses unique physicochemical properties, offering specific advantages and limitations depending on the intended therapeutic application. Studies suggest that the selection of an appropriate nanocarrier should be guided by the physicochemical characteristics of the drug, the nature of the target tissue, and the desired release kinetics [173, 176].

As observed in the pre-clinical studies in this review, specific limitations of the different antibody-conjugated polymer nanoparticles depend less on the type of polymer nanoparticle and more on the nanoparticle formulation and experimental parameters. For example, the method of antibody conjugation and the nature of the linkages formed can significantly impact the nanocarriers’ physicochemical properties, such as surface charge, antibody orientation, and antibody density, which in turn affect their behavior in both in vitro and in vivo models [177, 178]. Experimental conditions such as the pH influences the rate and extent of drug release from antibody-conjugated polymer nanoparticles. The targeted nanocarriers are designed to only exhibit higher drug release and tumor inhibition effect compared to free anti-cancer drugs, at a mildly acidic tumor microenvironment (low pH) [179, 180]. Furthermore, significant differences in therapeutic outcomes between antibody-conjugated nanoparticles, non-targeted nanoparticles, and free drugs were sometimes only observed during shorter treatment durations or exposure times. This may be due to the enhanced BBB penetration and cellular uptake facilitated by antibody targeting [169]. However, with prolonged exposure, non-targeted nanoparticles or free drugs may also accumulate in tumor tissues, diminishing the relative advantage of targeted systems [169, 181]. Lastly, the choice of cell line is a key factor, as the expression level of the target antigen varies across different cancer cell lines, thereby influencing the binding affinity and uptake efficiency of antibody-decorated nanocarriers [157].

### Polymeric micelles

Polymeric micelles are self-assembled block copolymers with a hydrophobic core and hydrophilic shell [182]. The hydrophobic core is formed from the assembly of hydrophobic segments of the nanoparticle while the hydrophilic segments are exposed on the outer region of the nanoparticle [182] The most commonly used techniques for polymeric micelle synthesis include direct dissolution [183], where amphiphilic polymers are dissolved in aqueous solution at concentrations above their critical micelle concentration (CMC); solvent evaporation, in which the polymer is first dissolved in an organic solvent, followed by evaporation of the solvent to form a thin film that is subsequently hydrated with water to induce micelle formation; and emulsion-based methods, which involve dissolving the polymer in a water-immiscible organic solvent, emulsifying this solution in water before removing the solvent via evaporation or dialysis for micelle assembly [184, 185].

Polymeric micelles, composed of amphiphilic block copolymers, are particularly effective for solubilizing hydrophobic drugs due to their core-shell structure. Their self-assembly in aqueous environments facilitates a relatively straightforward synthesis process compared to other nanoparticle types, enabling efficient transport of active compounds through the bloodstream and across the BBB [185]. However, micelles are prone to dilution-induced disassembly once their concentration falls below the CMC, leading to premature drug release in systemic circulation [185].

Some common monomers used include polyesters, polyethers and poly(amino acids), which can include PGA and PLA. The variability in the functional groups allows the conjugation and encapsulation of various drug compounds [185].

Polymeric micelles have high stability both in vitro and in vivo and good biocompatibility with the human body, which is essential to achieve in vivo therapeutic effects. They are often used to enhance the solubility of poorly soluble active agents and increase drug permeability and retention at target sites. When conjugated with antibodies, immunomicelles are formed [186]. A study demonstrated that conjugation with anti-transferrin receptor antibody (OX26) allowed an increase in in vivo cellular uptake of micelles into C6 glioma cells compared to the control group based on fluorescence studies. After 2–12 h of administration, there was a significant increase in the uptake of OX26-M(RhB) by glioma cells compared to micelles only labelled with RhB [152]. Another study developed polyethylene glycol (PEG)-based micelles conjugated with CD163 mAbs. The antibody supported the function of target tumor-associated macrophages for increased targeting specificity and tumor inhibition rate. Results from in vitro studies highlighted a more rapid and greater drug release from the PEG-based micelles under weak acid conditions at pH 5.0 and 6.0 compared to normal physiological pH, which suggests that the drugs can be released under acidic tumor microenvironment. In in vivo studies, a higher tumor inhibition rate with CD163 mAb conjugation was seen at 81% compared to unconjugated micelles and free drug, at 70% and 38.4% respectively, after 7 consecutive doses of the treatment [157].

Table 1 shows the summary of antibody-conjugated polymeric micelles for brain cancer treatment found in the literature.

**Table 1:** Antibody-conjugated micelles for brain cancer treatment

Type of polymer used	Antibody-conjugated + target receptor	Encapsulated compound	Size	Surface charge	Outcomes (in vitro)	Outcomes (in vivo)	Reference
Maleimide-Poly(ethylene glycol)-block-Poly(lactic acid) (mal-PEG-b-PLA) and Methoxy Polyethylene Glycol-block-Poly(lactic acid-co-Dihydrocarvone/Rhodamine B) (mPEG-b-P(LA-co-DHC/RhB))	Anti-transferin (TfR) antibody (OX26) targeting transferrin receptors expressed on glioma cells (C6 cells)	Rhodamine B (RhB) fluorescent dye	50 nm	-	-Higher cellular uptake of OX26 conjugated micelles compared with control in brain tissue of rats after 2 h	-Higher rate of tissue delivery with OX26 conjugated micelles compared with control -Higher brain volume distribution at 12 h with conjugated micelles -Higher brain permeability surface area with conjugated micelles	[152]
PEG	CD163 mAb targeting tumor-associated macrophages (TAMs)	DOX	-	-	-Higher drug release in acidic tumor microenvironment with antibody-conjugated micelles compared to control -Higher inhibitory effect in M2-type macrophages compared to H22 cells due to higher expression of CD163 receptors -No significant difference in cellular uptake of antibody-conjugated nanoparticle compared to non-targeted nanoparticle after 1 h of incubation (difference only observed after 5 h)	-Higher tumor inhibition rate (81%) with antibody-conjugated micelles compared with micelles (70%) and free DOX (38.4%) after 7 consecutive doses -Higher TNF-α secretion in necrotic sites with antibody-conjugated nanoparticles compared to non-targeted nanoparticles and free DOX	[157]
1,2-Distearoyl-sn-glycero-3-phosphoethanolamine-N-(polyethylene glycol)) (DSPE-PEG)	Antinucleosome antibody (mAb 2C5) targeting tumor cells	Procalcitonin (PCT)	nm	-11.86 mV to + 6.4 mV	-Higher cytotoxicity with pH-sensitive mixed micelles (20% cell viability) compared to non-pH-sensitive mixed micelles (40% cell viability)	-Increased 4T1 cellular uptake of 2C5-conjugated micelles (geometric fluorescence mean of about 300%) compared with unconjugated micelle (geometric mean of about 150%)	[187]
Polyethylene Glycol-Polyethylene Glycol-Phosphoethanolamine (PEG-PE)	Antibody single chain fragment variable (scFv), targeting GLUT1 expressed in glioma cells	DOX and CUR	14.8 nm	-4.4 mV	-Significantly higher cellular uptake of 0.05-mol% GLUT-1 scFv-targeted micelles by U87MG cells compared with non-targeted micelles -Lower cellular uptake for 0.1-mol% GLUT-1 scFv micelles due to steric hindrance -No significant reduction in IC50 with GLUT-1 scFv compared to non-targeted micelle and free DOX after 48 h of incubation -Higher cytotoxicity with antibody-conjugated micelle with CUR (30% decrease in IC50) compared to control groups	-Higher tumor penetration into tumor with GLUT-1 scFv-targeted micelles -Higher cytotoxicity against U87MG cells with GLUT-1 scFv-targeted micelles (70%) compared to non-targeted micelle and free drug (35%)	[169]
N-(2-hydroxypropyl)methacrylamide	Anti-ganglioside (GD2) mAb, (scFv) targeting GD2 antigen-positive tumor cells	DOX	-	-	-Higher cytotoxicity against GD2-expressing cells with scFv-conjugated nanoparticle compared to non-targeted nanoparticles	-Increased antileukemia efficacy with antibody-conjugation than without	[188]
PLA-PEG	3F8 mAb targeting GD2 expressed on neuroblastoma cells	SN-38	272 nm	-22.8 mV	-Higher potency or lower IC50 values for 3F8-NPs compared with control groups -Higher potency with free SN-38 in 3D tumor models that may act as diffusion barriers to NPs	-Higher tumor-killing effect and survival with 3F8-NPs than non-targeted nanoparticles and free irinotecan in GD2-high tumor model -Higher tumor penetration with 3F8-NPs compared with non-targeted micelle or free drug, based on rhodamine fluorescence intensity -No significant difference in survival with 3F8-NPs and non-targeted nanoparticles in GD2-low tumor model	[149]
Polycaprolactone-PEG(PCLn-PEG)	Anti-EGFR antibody (EGa1), targeting epidermal growth factor receptor (EGFR) expressed in head and neck squamous cells	meta-Tetra(hydroxyphenyl)chlorin (mTHPC)	17 nm to 45 nm	-2.3 mV to -1.9 mV	-Higher drug retention time in micelles than control group -Significantly higher cellular uptake by A431 cells with EGa1-conjugated micelles than with HeLa cells with low EGFR expression -Higher cellular uptake with EGa1-P23 micelles compared to EGa1-P15 after 7 h due to optimal molecular size, whereas no uptake enhancement with EGa1-P9	-Higher circulation time with EGa1-conjugated micelles (45% ID) 4 h after injection compared to free micelle (17% ID) -No significant difference in clearance rates between EGa1-conjugated P23 micelle and unconjugated micelle	[177]
poly(ethylene glycol)-poly(aspartate-hydrazide-epirubicin)	Anti-PD1 antibodies targeting CT2A and GL261 glioma cells to stimulate immunogenic cell death	Epirubicin	30 nm	-	-Higher levels of immunogenic cell death with Epi/m -Resistance against aPD1 observed with PTEN-negative CT2A cells -Synergistic anti-tumor effects observed with Epi/m and aPD1 antibody (higher CD8 + T-cels recruitment to tumor sites)	-Higher permeability and tumor accumulation with Epi/m + aPD1 (160 times more) compared to free Epi and aPD1 alone	[189]
Azide-functionalized Polyethylene Glycol-b-Polyaspartic Acid (N3–PEG-b-PAsp) and Azide-functionalized Polyethylene Glycol-b-Poly-L-lysine (N3–PEG-b-PLL)	Fabs targeting EphA2 receptor expressed on PC3 cancer cells	Alexa Fluor 555 dye	30 nm	-	-Higher cellular uptake of Fab-PIC micelles (about 5 times higher) than free mAbs and targeted mAbs -Fab–PIC micelles exhibit higher, sustained cellular uptake via EphA2-mediated endocytosis compared to mAbs and Fabs		[190]
PLA	Anti-neu/anti-CD40 antibodies targeting dendritic cells and T-cells to stimulate anti-tumor response against RNEU + tumors	-	-	-	-No binding of anti-CD40 conjugated nanoparticles to TUBO cells (CD40 negative) -Stimulation of IL-12 secretion with anti-CD40 NP and dual targeted anti-neu/anti-CD40-NP compared to free anti-neu mAb and anti-neu NP	-Higher antitumor response, Th1 immune response, activation of cytotoxic T cells and NK cells observed with dual targeted anti-neu/anti-CD40-NPs than anti-neu and anti-CD40-NPs alone	[175]
PMLA	Anti-TfR mAb targeting transferrin receptor expressed in BBB	Antisense Oligonucleotides (AONs) targeting epidermal growth factor receptor (EGFR) and CK2α	-	-	-Higher reduction in CK2α expression by nanobioconjugates with AONs in LN229 and U87MG cells	-Higher survival rates among xenogeneic glioblastoma mice models with P/Cetu/CK2α, P/Cetu/EGFR, or P/Cetu/EGFR/CK2α compared with control group. Greatest prolongation in survival with P/Cetu/EGFR/CK2α. (increase in median survival time to 75 days in LN229-bearing mice and 56 days in U87MG-bearing mice)	[191]

### Dendrimers

Dendrimers are highly branched, monodisperse synthetic macromolecules with a tree-like architecture that enables precise control over size, shape, and surface functionality. This hyperbranched structure comprises monomers built around a central core, interior branching units, and terminal functional groups [192]. Dendrimers offer distinct advantages such as enhanced solubility of hydrophobic drugs, controlled release, and the ability to be functionalized with multiple ligands for targeted delivery [193]. The three domains that make up a dendrimer are the core, the branching layer, and the exterior functional groups. Their size can range from 1 to 100 nm [194]. Dendrimers have a low viscosity and high solubility, making them favorable options for drug delivery as they can be circulated in the bloodstream for longer [195]. Two main methods can be used to synthesize dendrimers: the divergent approach and the convergent approach [196]. Drugs can be encapsulated within the dendrimer branches or conjugated at the terminal functional groups by covalent or electrostatic bonds. As the interior of the dendrimer cavities can be modified to be hydrophilic, hydrophobic drugs encapsulated in them can be made soluble [196]. Dendrimers can be made hydrophobic or hydrophilic by modifying their surface characteristics and functional groups. They can be synthesized from various monomers, including amino acids, sugars, and polypeptides. The most common dendrimers are derived from PAMAM, which is hydrophilic and biocompatible [196]. While their structural tunability makes them highly customizable carriers, this often means that their synthesis is complex as it involves multiple iterative reaction steps that can hinder large-scale production [192].

Yang et al. developed PAMAM dendrimers conjugated with anti-EGFRvIII mAbs (L8A4) or anti-EGFR mAbs (cetuximab). The dual-targeting approach resulted in higher tumor accumulation and longer survival than single-antibody groups without antibody-conjugation. Biodistribution studies demonstrated that the mixture of C225 and L8A4-conjugated dendrimers administered via Convection-Enhanced Delivery (CED) resulted in 61.4% of the injected dose per gram (ID/G) being localized in the tumor. This was about 2 times higher than the ID/g for L8A4-micelle (30.8%) and C225-micelle alone (34.7%). Moreover, rats bearing composite tumors treated with both mAbs had a mean survival time of 55 days, which was statistically significantly longer (*p* < 0.0001) compared to rats treated with either BD-L8A4 (36 days) or BD-C225 (38 days) [197]. Another study investigated how CD133 conjugated dendrimers can be used to enhance boron neutron capture therapy (BNCT) targeted at radio and chemo-resistant glioma stem cells (GSCs). Results showed that polyamide amine dendrimer conjugated with CD133 encapsulating mercaptoundecahydrododecaborate (PD-CD133/BSH) resulted in significantly higher uptake in CD133 + cells (91.8%) compared to CD133 − cells (29.4%). Furthermore, mice with CD133 + SU2 gliomas treated with PD-CD133/BSH and administered with BSH for BNCT had the longest mean survival time (MST) of an average of 61.8 days compared to those that received only neutron radiation and those that received PD-CD133/BSH without BNCT [172]. A summary of antibody-conjugated dendrimers used for brain cancer treatment is listed in Table 2.

**Table 2:** Antibody-conjugated dendrimers used for brain cancer treatment

Type of polymer used	Antibody-conjugated target receptor	Encapsulated Compound	Size	Surface charge	Outcomes (in vitro)	Outcomes (in vivo)	Reference
PAMAM dendrimer	Anti-EGFRvIIImAb (L8A4) and anti-EGFR mAb (cetuximab)	Boron-10	-	-	-L8A4 and cetuximab bind to different parts of the EGFRvIII molecule	-Higher drug accumulation at the tumor site for the dual-antibody-conjugated dendrimer (61.4% of the injected dose per gram) compared with L8A4 conjugation (30.8%) and C225 conjugation alone (34.7%) -Higher mean survival time in rats with F98 gliomas (55 days) compared to L8A4 (36 days) and C225 alone (38 days) -Poor delivery of boron for BNCT with C225 mAb in EGFRvIII- expressing gliomas	[197]
PAMAM dendrimer	CD133 monoclonal antibodies targeting CD133-positive glioma stem cells (GSCs)	Boron-10	-	-	-Higher cellular uptake for CD133-conjugated dendrimers in CD133 + GSCs (90%) compared with in CD133- cells (40%)	-Higher boron accumulation in CD133 + glioma xenografts compared with CD133- xenografts after administration of PD-CD133/BSH with minimal accumulation in other sites -No significant difference in boron levels between CD133 + and CD133- xenografts with combination therapy (PD-CD133/ BSH and BSH) due to loss of selective targeting -Higher mean survival times in mice with CD133 + implanted glioma xenografts (61.8 days) compared with mice implanted with CD133- glioma xenograft (46.7 days)	[172]
PAMAM dendrimer	Anti-EGFR mAb, cetuximab (C225) targeting EGFRvIII mutant receptor	Methotrexate	-	-	-Higher in vitro mean nanoparticle accumulation in mutant F98 glioma cells (62.9% ID/g tumor) compared with F98 wild-type cells (11.3%/g tumor)	-No significant difference in median survival times, with C255-conjugated nanoparticle, free mAb and free drug, in glioma-bearing mice	[198]
Polylysine G5 dendrimers	anti-programmed cell death ligand 1 (aPD-L1) monoclonal antibody targeting programmed-death ligand 1	Cy5	21.4 nm	+ 21.1 mV	-Higher tumor penetration with G5-R nanodots	-Higher survival times in mice with CT26 tumors when aPD-L1 conjugated G5-dendrimers (> 50 days) compared with control groups (35 days)	[199]
Phosphorus dendrimer (AK128)	Anti-PD-L monoclonal antibody targeting programmed-death ligand 1	BSA	228.7 nm	-10.3mV	-Higher cellular uptake with dendrimer (2.2 times) than without dendrimers -No significant increase in NK cell proliferation with aPD1 or aPD1-conjugated dendrimer compared to AK128 alone -Highest BBB penetration efficacy with AK128-aPD1@M1m NCs compared with other groups -Higher levels of apoptosis of C6 cells when antibody-conjugated dendrimer was administered (31.7%) compared to control groups due to higher natural killer cell proliferation	-Dual targeting with M1m and aPD1 antibody allows enhanced recruitment of T lymphocytes and NK cells into glioma and spleen and increase in Tregs in tumor sites -Higher tumor inhibition efficacy with aPD1 conjugated dendrimer (11.02%) compared to control groups	[200]
Hyperbranched polymeric (HBP) nanoparticle (with PEGylation)	HER3 bispecific-antibody fragment, lumretuzumab, targeting HER3 receptor expressed in breast cancer brain metastasis	DOX	480 nm	-	-High targeting specificity of HER3-bsAb to HER3 receptors -Lower accumulation of HER3-HBPs in the brain than untargeted HBPs due to poorer BBB penetration as a result of larger molecular size	-No significant reduction in tumor growth with HER3-HBP-DOX group compared with untargeted group -More significant cytotoxicity for HER3-HBP than without antibody conjugation (14 times higher intracranial drug accumulation) with HER3-HBP than without antibody conjugation	[184]
Targeted Lyposome/activated nanoparticles (tLyp)	Anti-NKG2A monoclonal antibody targeting NK and T cells to stimulate immune response (also conjugated with cell penetrating peptides tLyp-1 to form tLyp/aNKNP-siRNA	siLSINCT5 (siRNA)	103.42 nm	6.81 mV	-More significant tumor growth inhibition with tLypNP-siRNA and tLyp/aNKNP-siRNA compared with non-targeted NP, but no significant difference seen between aNKNP-siRNA and NP-siRNA - Higher U87 cell apoptosis with tLyp/aNKNP-siRNA (38.94%) compared with control groups	-Higher U87 tumor growth inhibition with tLyp/aNKNP-siRNA than in control groups -Higher levels of NK and T cells with tLyp/aNKNP-siRNA than in control groups, as shown in immunofluorescence studies -Higher drug accumulation in brain tumor site with tLyp/aNKNP-siRNA compared to other groups 24 h post-injection, but this decreases 36 h after injection (metabolic activity)	[201]
G7-anti-programmed cell death ligand 1(G7-aPD-L1)	Anti PD-L1 immune checkpoint inhibitors		27.4 nm	-	-Significant increase in IL-2 production with G7-aPD-L1h conjugates (1.9 times higher) than untargeted nanoparticles -Higher cellular retention in cancer cells with G7-c conjugates than free aPD-L1h -Higher cytotoxicity with antibody conjugation than with untargeted nanoparticles, where no cytotoxic effect was observed	-Higher tumor accumulation of G7-aPD-L1 compared with non-targeted nanoparticle 72 h post-injection	[202]
PAMAM dendrimer-based polyplex containing plasmid DNA encoding the interferon-beta (PAMAM-R/pORF-IFN-β polyplex )	No antibody conjugated	human interferon beta (IFN-B) plasmid	-	-	-	-Higher tumor inhibition with PAMAM-R/pORF-IFN-β compared to without gene in mice with brain tumors -Higher tumor cell apoptosis and selectivity	[203]
Hyperbranched polyglycerol-conjugated poly(lactic-co-glycolic acid)- (OX26–HPG–PLGA)	Transferrin antibody (OX26), targeting transferrin receptors	Endomorphins (analgesic drug)	170 nm	-27 mV	-Uptake of nanoparticles into brain microvascular endothelial cells in a time- and concentration-dependent manner, with filipin enhancing uptake at the same NP concentration	-Stronger and earlier analgesic effect with OX26-conjugated nanoparticles compared with untargeted nanoparticles and free drug (higher uptake of nanoparticles into brain microvascular endothelial cells)	[166]

### Nanospheres

Nanospheres are polymer nanoparticles with a solid core and a polymeric matrix surrounding the core. The encapsulated drug is freely and evenly dispersed within or attached to the matrix. Their size ranges from 10 to 200 nm [204]. Made from biodegradable or non-biodegradable polymers, they offer controlled or rapid drug release depending on matrix modifications. Various synthesis methods are applicable for nanospheres, including polymerization, where monomers are polymerized directly within a dispersed phase to form uniform nanospheres and solvent displacement, in which a polymer solution is mixed in a water-miscible organic solvent with an aqueous phase [205]. Another common technique used is solvent evaporation wherein the polymer and drug are first dissolved in an organic solvent which is then removed via evaporation to produce nanospheres [205, 206]. Nanospheres can act as a system for the controlled release of drugs to the tumor site, while rapid release can be achieved via modification of the polymeric matrix [204].

When compared to softer carriers like micelles or nanogels, polymer nanospheres exhibit superior structural stability under physiological conditions. This stability supports sustained drug release, a critical feature for ensuring efficient delivery across the BBB and accumulation at brain tumor sites [207]. However, nanospheres generally have a lower surface-to-volume ratio than dendrimers, which can limit the extent of antibody conjugation for targeted delivery [208].

Chu et al. successfully developed a novel multi-drug loaded antibody-conjugated polymeric nanoparticle system designed to overcome glioblastoma resistance against conventional chemotherapeutics [209]. A complex system consisting of a polymerizable monomer of TMZ in the form of temozolomide methacrylate (TMZ-MA), dialdehyde O^6^ benzylguanine (DABG), PEGMA, EGDMA conjugated with Cy5 and an αPEG-αEphA2 bispecific antibody (αEphA2 BsAb). The incorporation of DABG as an O^6^ alkylguanine DNA alkyltransferase (AGT, DNA repair protein) inhibitor conjugated using an acid-labile hydrazone linker, enabling controlled release under conditions encountered within the tumor microenvironment, serves to overcome potential resistance to TMZ. Meanwhile, αEphA2 BsAb was conjugated with the PEG components in the polymeric system, whereby the αPEG binding domain specifically recognizes and binds to the PEG antigens on the polymeric system, while the αEphA2 binding domain recognizes the ephrin type-A receptor-2 (EphA2) which is a specific surface receptor highly expressed on the surface of GBM cells and glioma stem cells. Through this system, the authors successfully controlled the amount and proportion of therapeutic agents being delivered within the polymeric system with great precision. Meanwhile, the use of an αEphA2 BsAb further allows for improved selectivity in targeting cancer cells. Findings from this study revealed an improved IC50 against T98G cell line when using TMZ with DABG (99 ± 9 µM) compared to TMZ (380 ± 87 µM) and DABG (125 ± 77 µM) alone. In vitro flow cytometry on T98G (glioblastoma) and U87MG cells and confocal microscopy imaging on T98G cells revealed selective uptake and accumulation of αEphA2 BsAb conjugated drugs-loaded polymers into cells that express the EphA2 receptor. Furthermore, the authors studied the selectivity of their system using a dual-tumor bearing xenograft murine model hosting human GBM cell lines U251 and U87MG with high and low expressions of EphA2 receptor, respectively. In this model, greater accumulation of the nanoparticles in U251 tumors compared to U87MG tumors on average was demonstrated, further enforcing the potential of this system for selective targeting. Although formulating this polymeric system capable of delivering precise doses of both TMZ and DABG posed notable synthetic challenges, this study underscores the promise of such dual-drug systems in targeting drug-resistant glioblastoma. The approach offers a valuable foundation for future refinement and development of more effective combination therapies [209].

Anti-TfR antibody-conjugated PMLA-PEG nanoconjugate with encapsulated TMZ was developed by Patil et al., demonstrating improved targeting specificity. Anti-TfR mAb conjugation to the nanoconjugate improved its potency, with antibody-conjugated PMLA-PEG nanoconjugate showing the strongest cytotoxic effect across U87MG, T98G, MDA-MB-231 and MDA-MB-468 cell lines [210]. Another study showed increased cellular uptake and reduced tumor cell viability in PLA-based nanoconjugates with Cetuximab attached to their surface. Results show enhanced intracellular drug bioavailability, as evidenced by significant reductions in cell survival after just 6 h of incubation with drug-loaded nanoconjugates, compared to free Alpelisib. This was achieved through the induction of apoptotic cell death. Overall, Cetuximab-conjugation improved intracellular drug uptake and allowed the slow drug release, targeting PI3K pathway inhibition [211]. More examples of antibody-conjugated nanospheres are listed in Table 3.

**Table 3:** ^Antibody−conjugated nanospheres for brain cancer treatment^

Type of polymer used	Antibody conjugated + target receptor	Encapsulated Compound	Size	Surface charge	Outcomes (in vitro)	Outcomes (in vivo)	References
PMLA and PEG	Transferrin (TfR) antibody targeting transferrin receptor on brain cancer cells and vascular endothelium	TMZ (TMZ)	6.5 nm to 14.8 nm	-6.3 mV to -17.7 mV	-Lower potency on U87MG cells with nanoconjugates than with free TMZ	-Increase in the half-life of conjugated TMZ (about 3–4 times) compared with free TMZ -In U87MG cells (TfR-expressing), free TMZ was more potent than nanoconjugate. The most potent nanoconjugate was P/PEG(2%)/LLL(40%)/TMZH(17%)/HuTfR mAb. -In MDA-M8-468 cells, nanoconjugates were more effective at reducing cancer cell viability than free TMZ	[210]
PLA	Cetuximab inhibiting the PI3K pathway	Alpelisib and,1’-Dioctadecyl-3,3,3’,3’-tetramethylindocarbocyanine perchlorate (DiR) dye	100 nm	-	-Reduction in cell viability (73.8%) for antibody-conjugated nanoparticles compared with non-targeted nanoparticles (70.6%) when tested in SCC cell lines at 6 h -Improved cellular uptake by Cal_33_ cells with cetuximab-conjugated nanoparticle (more than 85% of cells after 6 h) than non-targeted nanoparticles, based on fluorescence studies	-Improved tumor targeting with antibody-conjugated nanoparticles than without	[211]
Chitosan grafted with PEG	Transferrin (TfR) (CD71) antibody conjugation targeting transferrin receptors on brain cancer cells	Dil fluorescent dye	284.1 nm	+ 34.4. mV	-Higher cytotoxicity in hCMEC/D3 cells with TfRmAB-conjugated nanoparticles (77.3% cell viability) than with non-targeted nanoparticles (92%) after 24 h incubation -Higher cellular uptake in hCMEC/D3 cells than non-targeted nanoparticle -Internalization of TfRmAb-conjugated nanoparticles inhibited by both amiloride and chlorpromazine (inhibition of macropinocytosis and transcytosis)		[212]
PLGA	Anti-EGFRvIII MAb targeting EGFR receptors on glioblastoma cell line (DKMG/EGFRvIII cells)	Curcumin	249 nm to 280 nm	-13.1 mV to -1.4 mV	-Cytotoxicity for nanoconjugates is dose-dependent -More significant cytotoxicity towards DKMG/EGFRvIII glioma cells with MAb-CUR-PLGA NPs compared with non-targeted nanoparticles (at 40 and 60 µM) -Higher internalization in DK-MG^low^ cells of MAb-CUR-PLGA NPs than non-targeted nanoparticles -No cytotoxicity effects seen in DKMG/EGFRvIII cells with CUR-PLGA NPs and MAb-CUR-PLGA NPs without irradiation		[213]
Poly (butyl cyanoacrylate) (PBCA) with PEGylation	Anti-endothelial growth factor receptor (anti-EGFR) antibodies targeting EGFR expressed in glioblastoma cells	Carboplatin	365 nm	-10.7 mV	-Higher stability of drug when encapsulated in antibody-conjugated nanoparticle	-Higher survival times (23.5 days) in rats that were administered antibody-conjugated nanoparticles -Lower cytotoxicity to other tissues for antibody-conjugated nanoparticle than free carboplatin (40% higher)	[214]
PMLA	Anti-mouse transferrin receptor antibody and anti-nucleosome antibody 2C5 targeting receptors on U87MG glioma cels	Polycefin variant	20 nm to 30 nm	-	-Higher cytotoxicity with antibody-conjugation than without -Highest U87MG cellular uptake of 2C5-conjugated nanoparticle after 1 h. No internalization without 2C5 antibody.	-Higher drug accumulation in cultured human glioma cells and tumors in nude mice with induced glioblastoma with dual-targeted nanoparticle compared with nanoparticles only conjugated with anti-TfR or 2C5 antibody	[174]
Chitosan-PLGA	Cetaxizumab (CTX) antibody targeting different glioma cell lines (U251 and SW1088)	alpha-cyano-4-hydroxycinnamic acid (CHC)	213 to 875 nm	+ 33.2to + 58.9 mV	-Insignificant cell viability reduction when CHC is encapsulated in LGA/OCS NP, compared with PLGA/TMC NP -Higher cytotoxicity towards U251 and SW1088 glioma cells with antibody-conjugated nanoparticles compared to control (about 5% cell viability) -Enhanced antiangiogenic activity of conjugated nanoparticles compared to those without		[215]
PLGA-PEG	Anti-EGFRvIII MAb targeting EGFR receptors on glioblastoma cell line (U87MG vIII cells)	DOX	196.5 nm	-5.05 mV	-Higher cellular uptake was shown by MAb-DXR-PLGA NP -Enhanced sustained drug release at pH 7.4 by MAb-DXR-PLGA nanoparticles compared to without -Higher cytotoxicity towards U87MG vIII cell line shown by MAb-DXR-PLGA NP compared with unconjugated nanoparticle antibody conjugation		[216]
PMLA	Anti-TfR mAb targeting Transferrin receptors on glioma cells (T98G and RG62 cells)	Antisense oligonucleotides (AONs)	2 nm	-5.2 mV	-Different nanoparticle composition affects membrane-disruptive ability (P/LLL was pH independent while P/LOEt nanoparticle was pH-independent) -Higher cytotoxicity with P/LOEt nanoconjugates at 0.15 mg/mL but higher concentrations (0.5 mg/mL) caused cell shrinkage. This was not seen with P/LLL nanoconjugates	-Higher tumor growth inhibition with LLL-nanoconjugate (about 2 times) than LOEt-nanoconjugate -No anti-tumor effect with nanoconjugates without AONs or anti-TfR mAbs -Reduction in brain vessel area with LOEt/AON/Hu/Ms and P/LLL/AON/Hu/Ms than in control group	[180]
PLGA	Anti-OX40 mAb to enhance cytotoxic T-cell (CD4 and CD8) response against glioma cells	Anti-OX40 mAb	86 nm	-12.8 mV	-Higher sustained release of anti-OX40 mAb compared to control -Increased cytotoxic activity of cytotoxic T-cells with anti-OX40-PLGA-NP -Higher CTL proliferation with anti-OX40-PLGA-NP compared with anti-OX40 mAb and untargeted nanoparticle -No significant difference in T-cell proliferation between anti-OX40-PLGA-NP and immobilized anti-OX40		[217]

### Nanocapsules

Nanocapsules consist of a core where drugs can be encapsulated and an outer coating or shell that increases drug stability and solubility while protecting the drug from degradation [218]. The outer shell can be made of various polymers, including polyglycolic acid (PGA), polylactic acid (PLA), and PEG(PEG). These nanocapsules help control the drug release rate from the core and improve drug bioavailability [219]. On the other hand, the inner core can be aqueous or oil-based, allowing encapsulation of both hydrophilic and hydrophobic drugs. Most polymeric nanocapsules have an oily core, which facilitates the dissolution of hydrophobic drugs, improving their therapeutic efficacy. Polymer nanocapsules can be synthesized using nanosuspension of nanoemulsion templates with organic solvents at the early stages [220].

Nanocapsules, characterized by a core-shell structure, enable high drug-loading efficiency and improved solubility of hydrophobic drugs. This property is similar to that seen with micelles. Nevertheless, their thin polymeric shell is susceptible to degradation, aggregation, and instability during synthesis, sterilization, and storage, posing challenges for scalability and shelf-life stability [221].

Table 4 summarizes some antibody-conjugated polymeric nanocapsules that are being tested in preclinical studies to treat brain cancer. Tsutsui et al. created hybrid bionanocapsules (BNCs) decorated with anti-EGFRvIII antibodies. Promising results were obtained as the BNCs successfully enhanced tumor targeting specificity and reduced cytotoxicity towards non-tumor tissues [222]. In vitro studies demonstrated that these hybrid BNCs were effectively delivered to Gli36 glioma cells but showed minimal uptake in normal rat astrocytes. Fluorescence microscopy revealed punctate red fluorescence in Gli36 cells incubated with 1.1 µg/ml hybrid BNCs, indicating successful internalization. In contrast, normal astrocytes exhibited negligible fluorescence under the same conditions. Similar imaging results were observed in vivo as hybrid BNCs were detected in brain tissue tumor, but not in normal brain tissue [222].

Another study demonstrated the potential of anti-TMEF-2 mAb in increasing drug accumulation at brain tumor sites, with enhanced efficacy compared to free drug, docetaxel (DCX). Key results include significant inhibition of cell proliferation with DCX-loaded-anti-TMEFF-2-conjugated nanoparticles in A549 cells, compared to free DCX or unconjugated nanoparticles at 24 h [223]. Additionally, an in vivo study using mice with A549 tumor xenografts showed that at the study endpoint (14 days post-treatment initiation), tumor weights in treated groups decreased from an average of 1.8 g in controls to approximately 0.8–1.0 g with the administration of DCX-loaded CS-PEG-anti-TMEFF-2 mAb nanoparticles [223].

**Table 4:** Antibody-conjugated nanocapsules for brain cancer treatment

Type of polymer used	Antibody conjugated + target receptor	Encapsulated Compound	Size	Surface charge	Outcomes (in vitro)	Outcomes (in vivo)	References
Bionanocapsules made of L protein (hybrid BNC)	Anti-EGFR antibody targeting mutant EGFRvIII expressed by Gli36 glioma cells	-	80 nm	-	-Higher BNC accumulation in Gli36 cells compared to that in normal brain cells or tissues -Fluorescence studies showed anti-EGFR antibodies localized on cell surface. BNCs not internalized via antibody-receptor interactions.	-Higher BNC accumulation in tumor site compared to that in normal brain cells or tissues	[222]
Chitosan-g-PEG nanocapsule (CS-PEG-anti-TMEFF-2 mAb NCs)	Anti-TMEFF-2 monoclonal antibody, targeting	Docetaxel (DCX)	200 nm	+ 27.1 mV to + 38.7 mV	-Reduction in cell proliferation rate with nanocapsules compared to free DCX after 24 h. However, no significant difference after 48 h between groups.	-No significant difference in tumor size reduction between targeted nanoparticles, non-targeted nanoparticles and free DCX at 14 days post-treatment -Poor penetration deep into tumor with mAb-functionalized nanoparticles -No significant toxicity effects to non-tumor tissue in all groups (bone weight loss < 20%)	[23]
Antibody affinity motif of protein A (Hybrid bio-nanocapsules)	Anti-EGFR antibodies, targeting mutant EGFR	7-propionyl taxol 2′-O-α-D-glucoside	-	-	-Higher drug loading capacity in hybrid nanoparticle (120-fold) with 7-propionyltaxol 2′-O-α-D-glucoside than with taxol		[223]
Chitosan-coated lipid-core nanocapsules (MLNC)	Bevacizumab targeting VEGF expressed in glioblastoma cells	Functionalized with PEPvIII (EGFRvIII targeting peptide) and gold ions	183 nm	+ 13 mV to + 19 mV		-Higher reduction tumor size with MLNC-PEPvIII and MLNC-BCZ nanoconjugates (87%) compared with the untreated control group -Higher CD8 + T lymphocytes stimulated with MLNC-PEPvIII and MLNC-BCZ nanoconjugates compared to untreated control group and group receiving free PEPvIII and BCZ	[224]

### Nanogels

Nanogels are three-dimensional, crosslinked polymer networks that possess swelling capabilities in the presence of water. Nanogels are synthesized from amphiphilic polymers like PLGA and PEG that self-assemble through physical or chemical processes, and have been used as a delivery system for chemotherapy agents that target tumors in cancer [225]. Another method of synthesis was to suspend them in a hydrogel matrix to create a hybrid system for structural support, increasing their stability in the body by preventing the degradation of nanogels [225, 226].

Nanogels can be engineered to have enhanced sensitivity to certain stimuli in the body such as pH and temperature, allowing drug release in an acidic tumor microenvironment in the body at 37 °C. Brain tumors like glioblastoma often exhibit a more acidic extracellular pH (6.5 to 7.0) than other tissues (7.4). In such conditions, functional groups in the nanogels polymers undergo protonation or deprotonation, leading to swelling and drug release [227]. Additionally, the pH can trigger the cleavage of cross-linkages in the nanogel polymers, facilitating the release of the drug at the tumor site. Liao et al. developed polymeric nanogels with benzoic-imide cross-linkages that were cleaved as pH falls from 7.8 to 6.4, allowing load release at tumor sites [228]. Nanogels are also capable of encapsulating bioactive compounds of various molecular weights, making them highly versatile. However, the presence of surfactants in nanogels has been associated with cytotoxicity, necessitating further refinement in their formulation [182].

Nanogels conjugated with hyaluronic acid enable DOX release via ester bond hydrolysis in low pH, enhancing stability and drug accumulation at tumor sites [229]. However, early swelling in vivo remains a risk, as even slight pH changes can cause drug release in non-target tissues. Variability in brain tumor pH across patients complicates precise tuning of nanogel pH sensitivity. Despite this, tumor pH differences may not significantly impact the overall therapeutic efficacy of the nanogels [230].

A study showed that anti-CC49 mAb-PEG-b-PMA nanogel can target antigens expressed by target tumor cells, particularly the glycoprotein 72. The experiment demonstrated how functionalization of nanogels can result in improved affinity and specificity for the target antigen, Bovine Submaxillary Mucin (BCM). Surface Plasmon Resonance (SPR) was employed to evaluate the antigen-binding specificity of the nanoconjugate. Results showed that unconjugated nanogels or those that were decorated with non-specific antibodies had negligible binding to the antigen [231]. Additionally, Liu et al. tested anti-CD47 antibody as a potential ligand for conjugation. Bone marrow-derived dendritic cells (BMDCs) and macrophages (BMDMs) exhibited increased phagocytosis of 4T1 cells pretreated with Dox@HFn, as evidenced by flow cytometry analysis [232]. Enhanced T-cell activation was also observed as co-culture of activated BMDCs with T cells resulted in significantly higher expression of CD69 on CD8 + T cells and increased secretion of cytokines IFN-γ and TNF-α. Higher in vivo drug accumulation and increased survival rates in glioblastoma mice were seen. Fluorescence imaging showed that Dox@HFn GelL exhibited an increasing fluorescence signal, while free DOX showed no detectable signal by day 5 [232]. Table 5 summarizes the different types of antibody-conjugated nanogels for brain cancer treatment.

**Table 5:** Antibody-conjugated nanogels for brain cancer treatment

Type of polymer used	Antibody conjugated + target receptor	Encapsulated Compound	Size	Surface charge	Outcomes (in vitro)	Outcomes (in vivo)	Reference
PEG-b-PMA	CC49 mAb targeting glycoprotein 72 expressed by tumor cells	-	158.9 nm	-17.0 mV	-	-	[231]
Ferritin	Anti-CD47 antibody against glioblastoma tumor	DOX and oxidized dextran	12 nm	-	-Higher tumor penetration, retention and accumulation in 4T1 cells with Dox@HFn Gel compared with Dox@HFn and free drug	-Higher survival rates in glioblastoma mice with antibody-conjugated Dox@HFn Gel (50 days) compared with the control group (34 days) -Higher median survival with Dox@HFn gel and anti-CD47 antibodies compared with control surgery group (50 vs. 34 days)	[232]

### Polymersomes

Polymersomes are vesicle-like structures formed by amphiphilic block copolymers, with a hydrophilic core and a thick polymeric bilayer membrane as the outer shell [150]. They can encapsulate hydrophilic drugs inside the core and hydrophobic drugs within the membrane. Commonly used polymers include PEG and PDLLA. The thick membrane that encapsulates the polymersomes provides stability and strength to the polymersomes compared to liposomes, which have a similar chemical structure and preparation methods, such as solvent switching technique and double emulsion [233]. Solvent switching involves dissolving amphiphilic block copolymers in an organic solvent before the addition of water to induce self-assembly [234]. The double emulsion method involves an additional step wherein two emulsifications are performed sequentially. After the primary emulsion is stabilized, it is dispersed into a hydrophobic organic phase to form a double emulsion before the solvent is removed [235]. Polymersomes have been modified to enhance stimuli-sensitivity so that drugs can be released with changes in pH or temperature for a more controlled drug release [236].

Polymersomes, which are bilayer vesicles structurally similar to liposomes, offer the dual capacity to encapsulate both hydrophilic and hydrophobic agents, unlike many polymer nanoparticles. Despite their versatility, limitations of polymersomes include low drug-loading efficiency and complex fabrication processes, which hinder large-scale manufacturing and clinical translation [18].

A study conducted by Tian et al. showed that polymersomes conjugated with Cys-Angiopep antibody enhanced targeting of BBB endothelial cells via interaction with the LRP-1 receptors, increasing drug delivery and increased cellular uptake via transcytosis and endocytosis processes [128]. Drug release was also enhanced in acidic tumor microenvironment. Nevertheless, polymersome targeting facilitated entry into endothelial cells but not across the BBB into the parenchyma, suggesting the need for further optimization of targeting strategies to enhance BBB penetration [24]. Additionally, a separate study highlighted that mPEG-PDLLA polymersomes with anti-EGFR antibodies as a targeting ligand demonstrated enhanced drug release at low pH due to its pH-sensitive properties. The study investigated the enzymatic degradation of the polymersome by cathepsin B (Cath B) at pH 5.5 and 7.4 using dynamic light scattering. There was a decrease in scattering intensity over 7 days at pH 5.5 due to the cleavage of the peptide linker of the polymersome. However, no degradation was seen at pH 7.4, indicating that polymersome dissociation is favoured at acidic pH. In terms of cellular uptake, polymersomes functionalized with anti-EGFR antibodies showed increased internalization into SKBR3 cells compared to unconjugated nanoparticles [237]. Further examples are highlighted in Table 6.

**Table 6:** Antibody-conjugated polymersomes for brain cancer treatment

Type of polymer used	Antibody conjugated + target receptor	Encapsulated compound	Size	Charge	Outcomes (in vitro)	Outcomes (in vivo)	Reference
Poly(2-(diisopropylamino)ethyl methacrylate) (PDPA)	Cys-Angiopep and Cys-RVG targeting LRP-1 receptor expressed by BBB endothelial cells	IgG	100 nm	-	-Enhanced drug delivery across the BBB with Angiopep-2-functionalized nanoparticle (LRP-1 mediated transcytosis) -Increased cellular uptake by bEnd.3 cells with antibody-conjugated nanoparticle compared to control group	-LRP-1 enhances pH-sensitivity of polymersomes to promote drug release in acidic tumor microenvironment	[128]
Methoxy poly(ethylene glycol) (mPEG) and poly(d, l-lactide) (PDLLA)	Anti-epidermal growth factor receptor (anti-EGFR) antibodies targeting EGFR expressed in cancer cells	Acridine orange (AO)	124 nm	-	-AO release from peptide-functionalized polymersome at low pH (5.5) over 3 days at tumor site. However, the release plateaued. -Increased cellular uptake of antibody conjugated polymersomes by SKBR3 cells compared to non-targeted polymersomes	-	[237]

## Challenges and limitations in clinical translation of antibody-conjugated polymer nanoparticles

### Scalability and manufacturing

Despite the use of various conjugation strategies to develop antibody-functionalized polymer nanoparticles, significant challenges continue to hinder their scalability and suitability for large-scale manufacturing. Antibodies are structurally complex biomolecules with multiple reactive functional groups, such as amines and thiols, which can lead to non-specific conjugation, resulting in variability in conjugation efficiency, antibody orientation, and retained biological activity [238]. To overcome these issues, site-specific conjugation strategies are often required to ensure uniform attachment, reproducibility, and preservation of antibody functionality, which are critical for achieving consistent and effective anti-tumor responses [148]. This typically entails more time-consuming and complex methods, such as enzymatic or tag-mediated conjugation systems.

Following conjugation reactions, purification steps such as filtration, precipitation, and ultracentrifugation are essential to remove unconjugated antibodies, excess reagents, and other undesired by-products, ensuring the integrity and specificity of the final antibody-functionalized nanoparticle formulation [148]. However, due to the inherent instability of antibodies, parameters such as temperature and pH must be tightly controlled to prevent degradation of the nanoformulation. Antibodies are particularly sensitive to certain solvents and mechanical stress, necessitating careful optimization of the process to preserve both the physicochemical integrity and therapeutic efficacy of the final product [239].

To enable specific targeting by antibody-conjugated nanoparticles, antibody design must be individually customized and optimized during manufacturing to recognize distinct blood–brain barrier (BBB) or brain cancer biomarkers [143]. This requirement introduces significant complexity into the development process, as each target biomarker may demand unique antibody affinities, binding kinetics, and structural considerations. As a result, extensive screening and validation are often necessary to ensure that the conjugated antibodies maintain their specificity and functional activity upon attachment to the nanoparticle surface [240, 241]. Moreover, this customization increases time, cost, and regulatory burden, as each formulation may require separate optimization protocols, stability assessments, and safety evaluations—ultimately limiting the scalability and commercial viability of such targeted therapies [242]. Furthermore, purification techniques commonly used at the laboratory scale, such as ultracentrifugation or affinity chromatography, are often challenging to adapt for large-scale manufacturing due to limitations in scalability, throughput, and cost-effectiveness. Sterilization and packaging must also be carefully managed to maintain the integrity of the nanomedicine products [241]. Given the relative instability of antibody-conjugated polymer nanoparticles, careful screening of excipients is essential to identify compatible dispersion buffers, stabilizers, and antioxidants that support long-term storage and maintain formulation integrity. Although extensive processes are required to achieve this, advanced tools like high-throughput screening and machine learning can be integrated to accelerate and optimize the process [148].

### Formulation

Another major challenge limiting the scalability of antibody-conjugated polymer nanoparticles is the inherent instability of antibodies. As antibody-based therapies are typically administered parenterally, the final nanoparticle formulations must be prepared in an aqueous form suitable for injection. However, the structural sensitivity of antibodies makes them prone to aggregation or denaturation over time, particularly in solution, where their stability is influenced by factors such as pH, temperature, and buffer composition [243]. To address this challenge, manufacturers rely on lyophilization, also known as freeze-drying, to preserve antibody colloidal stability during storage and transport [244]. This process involves freezing the product to solidify all the water content in the formulation, converting it into ice. The frozen formulation is later put under vacuum condition to remove the water through sublimation followed by desorption [245]. However, the process itself can introduce physical and chemical stresses, such as freezing-induced pH shifts, ice crystal formation, and protein adsorption at the ice interface, which may lead to irreversible nanoparticle aggregation and antibody denaturation. Therefore, optimization of the lyophilization process and selection of suitable cryoprotectants are crucial to maintain colloidal stability and functionality of the antibody-conjugated nanoformulation [246].

### Toxicity profile

The biosafety of antibody-conjugated polymer nanoparticles is a critical factor for their clinical translation. Potential cytotoxic effects, such as organ-specific toxicities, often arise from factors including the polymer’s composition, surface chemistry, surface charge, and nanoparticle instability [247, 248]. Conjugation chemistry can also be an important cause, as certain linkers and reactive groups can activate the complement system and consequently, negative side effects. One study demonstrated that thiol-maleimide chemistry can trigger complement activation through interactions between reactive maleimide groups and circulating albumin. This interaction led to protein aggregation and a significant increase in pulmonary phagocyte recruitment, revealing a previously underappreciated mechanism of immunotoxicity [25]. The immune system can also be activated by an azide-DBCO click reaction as a result of excessive aggregation of antibodies on the nanoparticle surface. This can lead to adverse effects such as inflammation and altered biodistribution [143]. Additionally, interactions between the Fc region of surface-bound antibodies and Fcγ receptors on immune cells can provoke antibody-dependent cellular cytotoxicity (ADCC), a well-documented phenomenon in the context of antibody–drug conjugates that may also occur with antibody-functionalized nanoparticles [249]. Thus, careful selection of the conjugation strategy, linkages used and the nanoparticle design is crucial to minimize adverse immune reactions.

## Research gaps and future directions

Overall, the majority of studies report promising outcomes for the use of antibody-conjugated polymer nanoparticles in the treatment of brain cancer. Antibody conjugation resulted in higher cellular uptake in glioma cells, higher BBB permeation and tumor penetration, enhanced antitumor activity, and reduced cytotoxicity towards non-target tissues, showing that these nanoformulations overcome challenges associated with conventional treatment methods.

Antibodies are an effective therapeutic agent on their own as they target certain molecules and cellular pathways involved in cell proliferation and metastasis of tumors. Some studies suggest that conjugating nanoparticles with more than one type of antibody or with other targeting ligands to target multiple brain tumor biomarkers can lead to higher drug accumulation and other therapeutic benefits [198, 250–252]. While there have been very few studies investigating dual-targeting polymeric nanoparticles for brain cancer, existing studies show encouraging results, including an increase in BBB penetration and higher tumor accumulation with reduced off-target effects [174, 175]. Comparable studies involving other nanoparticle formulations have likewise demonstrated encouraging outcomes. For instance, Kuo et al. developed solid lipid nanoparticles (SLNs) conjugated with 8314MAb and anti-EGFR antibodies (AEGFR). The dual-targeting nanoparticle enhanced BBB penetration via interaction with insulin receptors on brain endothelial cells and improved targeting of EGFR-expressing U87MG glioblastoma cells, leading to controlled etoposide (ETP) release and significant inhibition of tumor cell proliferation [253]. Furthermore, another study demonstrated that dual-targeting immunoliposomes functionalized with Angiopep-2 and CD133 mAb enhanced TMZ delivery across the BBB and specifically targeted glioma stem cells [251]. Hence, multi-targeting has a great space of expansion in terms of research in polymeric nanoparticles in targeting brain cancer.

Despite the overall promise, some investigations have identified limitations and adverse outcomes. Due to the highly aggressive nature of brain tumor, antibody-drug conjugation does not always result in improved therapeutic efficacy compared to administration of the drug or antibody alone. For example, no significant difference in mean survival time was observed between mice treated with the C225-G5-MTX cetuximab bioconjugate and those receiving methotrexate or cetuximab alone, despite favorable biodistribution profiles [198]. As treatment was initiated 7 days post-implantation, tumor burden may have already been substantial, potentially hindering effective drug accumulation at the tumor site, restricting the therapeutic window. These findings underscore the need for earlier intervention, particularly in fast-growing tumors, to maximize therapeutic impact. Further investigation into optimal treatment timing, dosing regimen, and nanoparticle pharmacokinetics is warranted. Again, incorporating ligands targeting multiple tumor-specific markers may enhance efficacy through improved tumor targeting.

Some conflicting findings were also reported, where highly conjugated ligand or antibody density in certain types of nanoparticles led to reduced cellular uptake or BBB penetration [253, 254]. This effect may be attributed to steric hindrance, which can limit interactions with target receptors [255]. Additionally, a high density of surface-bound antibodies may also impair the ability of nanoparticles to traverse the blood-brain barrier (BBB) due to increased particle size, altered surface properties, or altered uptake pathways of the nanoparticles into the BBB endothelium [254]. This is also supported by a study conducted by Heggannavar et al. using PCL nanoparticles conjugated with varying concentrations of transferrin achieved optimal U87 cellular uptake of paclitaxel at intermediate concentrations [254]. Thus, targeting-ligand density must be optimized based on the type of antibody used and tumor model to achieve improved outcomes in drug delivery to tumors. This is a research gap that must be explored to enhance the nanoformulation before proceeding to clinical trials to maximize patient outcomes [255, 256].

Notably, some in vitro models reported in the literature demonstrate careful design and methodological robustness. For instance, the use of 3D cancer cell spheroids of U87MG for in vitro studies to mimic the tumor microenvironment more closely compared to the commonly used 2D monolayer cultures; where 3D cancer cell spheroids allow for greater cell diversity to consider different cell behaviors in the brain tumor [257, 258]. Spheroids also establish gradients of oxygen and nutrients between cell layers as seen in in vivo tumors, allowing the replication of the hypoxic environment deep in tumors. This approach enables a more accurate assessment of nanoformulation performance regarding drug penetration and therapeutic response, thereby enhancing comparability with complex in vivo tumor models [257]. Future studies may consider adopting this in vitro model to strengthen the validation of their findings. Some studies successfully developed an effective model to mimic the brain environment, particularly the BBB, which is a major challenge for brain tumor treatment. A bi-culture Trans-well setup, where BBB endothelial cells were cultured, was utilized to compare the penetration extent of antibody-conjugated polymer nanoparticles across the BBB [259]. This approach provides a valuable platform for evaluating nanoparticle transport mechanisms and optimizing design parameters to enhance brain-targeted delivery. Nevertheless, while many studies have assessed the in vitro cellular uptake of nanoformulations by glioma cells, they often failed to incorporate models that simulate BBB penetration. This oversight limits the relevance of their findings, as it underrepresents the physiological barriers encountered in actual tumor conditions. Robust in vitro models that mimic the BBB are essential for bridging the gap between preliminary cellular assays and in vivo studies, ensuring that observed therapeutic effects are more likely to translate to animal or human models. The disparity in pre-clinical outcomes could also be attributed to a lack of standardization of in vivo models used. Some studies employed orthotopic brain tumor xenografts while some implanted tumor in nude mice; but there is no clear evidence on whether they can replicate human brain tumor, which is a major obstacle in brain cancer treatment. The inherent heterogeneity of brain cancer, coupled with the fact that some originates from the brain, while others occur as a result of metastasis further complicate study designs. This presents a significant challenge in preclinical studies, particularly in the use of varying cell lines ranging from brain cancer cells U87MG, T98G, LN229, U251, to 4T1 breast cancer cells and SCC lung cancer cells, all of which behave very differently. This inconsistent cell line selection hinders the translation of preclinical studies to human clinical trials as similar positive outcomes cannot be achieved. The diversity of brain cancer cell lines also hinders the standardization of preclinical studies, making direct comparison between studies extremely challenging in understanding the pharmacokinetics and toxicity of nanoformulations in humans.

Due to the absence of effective control groups, it is difficult to draw a clear conclusion about whether improvements in drug uptake and tumor inhibition in patients are due to antibody conjugation or the use of co-administered therapies and chemotherapy agents. Many studies combine different treatment approaches, either by encapsulating multiple drugs in antibody-conjugated polymer nanoparticles or by administering chemotherapy agents or radiotherapy alongside the nanoformulations. This approach allows researchers to explore the synergistic or additive effects of multiple drugs co-delivered in the nanoparticles as patients with brain cancer can respond differently to the same drug due to biological variations. Combination therapy can improve patient outcomes by targeting various cancer mechanisms and signaling pathways, helping to reduce tumor resistance and recurrence. Therefore, more comprehensive studies with proper controls are needed to assess the individual contributions of each therapy or drug in both the short and long term.

Most current research is primarily focused on evaluating the efficacy of nanoparticles for treating glioma, with a particular emphasis on GBM. Since positive preclinical outcomes have been reported for the use of antibody-conjugated polymer nanoparticles for glioma, it may be worth extending studies to include other types of brain cancer, including meningiomas, medulloblastomas, and CNS lymphoma although prevalence rates may be much lower than glioma. This allows for greater representation of the preclinical results across all types of brain cancers. Understandably, time, budget and geographical constraints may pose challenges to broadening the research scope. It is also costly to mass produce antibody-conjugated nanoparticles specific to different target receptors or other biomarkers for a widely heterogenous disease like brain cancer. However, with research collaborations and more funding opportunities, studies can be catered to underserved brain cancers.

While there are currently no clinical trials specifically investigating antibody-conjugated polymer nanoparticles for brain cancer treatment, recent clinical trials on different nanoparticles and antibody-based therapies for cancer provide promising insights. For example, a gadolinium-chelated nanoparticle, engineered for theranostic applications that combine radiosensitization with multimodal imaging capabilities, is currently being evaluated in conjunction with whole brain radiation therapy in patients with brain metastases, with tumor response being assessed at predefined intervals following treatment initiation (NCT number: NCT03818386).

Although polymer nanoparticles have not yet entered clinical trials specifically targeting brain cancer, their safety profile and therapeutic potential are currently being investigated in other cancer indications. Notably, a Phase II randomized trial is comparing the efficacy of two induction chemotherapy regimens, TPC (comprising paclitaxel formulated as polymeric micellar paclitaxel, cisplatin, and capecitabine) versus the standard GP regimen (gemcitabine and cisplatin), both administered alongside concurrent chemoradiotherapy in patients with high-risk, locoregionally advanced nasopharyngeal carcinoma. This trial aims to elucidate differences in 2-year progression-free survival, overall survival, tumor response rates, and treatment-related toxicities (NCT number: NCT06301165). Given the promising therapeutic potential of polymer nanoparticles, Genexol-PM—a PEG-PLA-based polymeric micelle encapsulating paclitaxel, which has been approved in South Korea for the treatment of breast, lung, and ovarian cancers [260]. A completed clinical study demonstrated the potential of ligand-directed polymeric nanoparticles, such as BIND-014, a PEG-PLGA nanoparticle engineered to target tumor cells overexpressing prostate-specific membrane antigen (PSMA) via a PSMA-binding ligand. While standard docetaxel chemotherapy remains a strong benchmark, BIND-014 was well-tolerated and showed encouraging clinical activity. Notably, a significant reduction in PSMA-positive circulating tumor cells (CTCs) was observed, indicating a favorable treatment response in PSMA-positive patients [261].

Multiple clinical trials are rigorously assessing the safety, tolerability, and therapeutic efficacy of ADCs across various brain cancer subtypes. Following the FDA’s 2019 approval of Trastuzumab-Deruxtecan (T-DXd) for HER2-positive breast cancer, a Phase II study has been initiated to evaluate the intracranial activity of this ADC in patients with HER2-mutant advanced non-squamous non-small cell lung cancer (NSCLC) who show asymptomatic brain metastases, with the primary endpoint being the median intracranial progression-free survival [54].

This ADC has already demonstrated robust clinical efficacy in HER2-positive breast, gastric, and lung cancers (NCT number: NCT06250777). Moreover, another Phase II trial is investigating the safety and efficacy of T-DXd in patients with HER2-low breast cancer presenting with active brain metastases (NCT number: NCT06048718). Additionally, a separate clinical trial is exploring a combination regimen consisting of Datopotamab-deruxtecan (an ADC), Carboplatin, and Pembrolizumab in metastatic NSCLC patients with brain metastases, with intracranial objective response rate serving as the principal measure of treatment efficacy (NCT number: NCT06822543).

To enhance the limitations of traditional ADCs (ADCs), polymer-based ADCs (pADCs) have been developed, in which the drug payload is linked to the antibody via a polymer scaffold. Unlike conventional ADCs, which are restricted to conjugating a limited number of drug molecules per antibody due to steric hindrance, pADCs enable significantly higher drug-to-antibody ratios by leveraging the polymer’s capacity as a multifunctional carrier [262, 263]. A study demonstrated that trastuzumab conjugated with a vinca drug derivative through the Fleximer polymer achieved a high drug-antibody ratio of 20:1, while maintaining strong antigen-binding affinity and exhibiting substantial tumor accumulation in multiple xenograft models [263]. Unlike traditional ADCs, the use of polymeric linkers in ADCs enhances pharmacokinetics and drug stability in the circulation as the linkers can be engineered to prevent premature degradation of the drug and thereby prolonging drug half-life [264]. Importantly, the configuration and chemistry of the polymer linker significantly influence the overall stability of the conjugate. For instance, a study showed that Amide-linked ADCs with two pendant 12-unit PEG chains demonstrated superior physical and chemical stability compared to ADCs with linear PEG linkers [265].

Besides pADCS, antibody-polymer conjugates (APCs) have been gaining attention as a versatile strategy to overcome limitations of traditional ADCs. APCs involve binding of antibodies to polymers rather than drugs. By leveraging the properties of polymers, APCs increase the drug-to-antibody ratio without compromising antibody solubility and stability and reduce aggregation in circulation [266]. Incorporating hydrophilic comonomers within the polymer backbone increases the solubility of APCs unlike in ADCs in which high drug payloads increase hydrophobicity. Kanjilal and colleagues esigned an APC where a targeting antibody was conjugated to multiple copies of a random copolymer composed of hydrophilic oligoethylene glycol (PEG) units. instead of using PEG as a simple spacer, the PEG segments are randomly incorporated within the polymer backbone to improve solubility and mask hydrophobicity. Results showed that this APC had superior cytotoxicity in HER2 + cell lines compared to the conventional ADC, Kadcyla, making APCs promising strategies for cancer treatment [267].

Taken together, the accumulating clinical evidence supporting the efficacy of both polymer nanoparticles and ADCs across various cancer types, including those involving brain metastases, underscores the potential of antibody-conjugated polymer nanoparticles as a promising and innovative therapeutic strategy for brain cancer. Emerging designs of next-generation ADCs, such as APCs and pADCs in preclinical development, demonstrate ability to overcome limitations of traditional ADCs. This highlights the transformative potential of antibody-conjugated polymer nanoparticles for treatment of a range of cancer types, including brain cancer.

## Conclusion

Most preclinical studies have demonstrated that antibody-conjugated polymer nanoparticles enhance tumor targeting, drug uptake, accumulation, and cytotoxicity against brain tumors, compared to non-targeted nanoparticles and free drugs in in vitro models. Similar outcomes such as enhanced drug uptake and anti-tumor activity were also reported in in vivo models, with the exception of some contradicting results where no changes or reduced therapeutic benefit of antibody-conjugation was reported due to the use of high antibody density and certain experimental conditions, such as pH, duration of exposure to treatment, and choice of cell lines. Future studies should focus on standardizing and refining the quality of in vivo models to better mimic the brain tumor microenvironment and to promote the translation of these studies in a clinical setting. In addition, co-encapsulation of more than one treatment agent and multiple surface antibody-conjugation present a great opportunity for research in this area. Extensive studies should also be conducted to evaluate the mechanism exploited by antibody-conjugated polymer nanoparticles to increase BBB crossing to reach tumor site, rather than merely focusing on the therapeutic outcomes of the nanoformulations on inhibiting tumor growth. As manufacturing and scalability remain among the greatest challenges to the clinical translation of antibody-conjugated polymer nanoparticles, more research is necessary to optimize formulation parameters and improve the robustness of freeze-drying processes to preserve nanoparticle integrity, antibody functionality, and overall product stability. Addressing these limitations and challenges are necessary for the bench to bedside advancement of safe and effective nanoformulations for brain cancer treatment.

## Data Availability

More information regarding the data presented in this review will be available upon request.
